# Structural Basis of HIV-1 Neutralization by Affinity Matured Fabs Directed against the Internal Trimeric Coiled-Coil of gp41

**DOI:** 10.1371/journal.ppat.1001182

**Published:** 2010-11-11

**Authors:** Elena Gustchina, Mi Li, John M. Louis, D. Eric Anderson, John Lloyd, Christian Frisch, Carole A. Bewley, Alla Gustchina, Alexander Wlodawer, G. Marius Clore

**Affiliations:** 1 Laboratory of Chemical Physics, National Institute of Diabetes and Digestive and Kidney Diseases, National Institutes of Health, Bethesda, Maryland, United States of America; 2 Protein Structure Section, Macromolecular Crystallography Laboratory, National Cancer Institute, Frederick, Maryland, United States of America; 3 Basic Research Program, SAIC-Frederick, Frederick, Maryland, United States of America; 4 Proteomics and Mass Spectrometry Facility, National Institute of Diabetes and Digestive and Kidney Diseases, National Institutes of Health, Bethesda, Maryland, United States of America; 5 AbD Serotec, MorphoSys AG, Martinsried, Germany; 6 Laboratory of Biorganic Chemistry, National Institute of Diabetes and Digestive and Kidney Diseases, National Institutes of Health, Bethesda, Maryland, United States of America; University of Zurich, Switzerland

## Abstract

The conserved internal trimeric coiled-coil of the N-heptad repeat (N-HR) of HIV-1 gp41 is transiently exposed during the fusion process by forming a pre-hairpin intermediate, thus representing an attractive target for the design of fusion inhibitors and neutralizing antibodies. In previous studies we reported a series of broadly neutralizing mini-antibodies derived from a synthetic naïve human combinatorial antibody library by panning against a mimetic of the trimeric N-HR coiled coil, followed by affinity maturation using targeted diversification of the CDR-H2 loop. Here we report crystal structures of the N-HR mimetic 5-Helix with two Fabs that represent the extremes of this series: Fab 8066 is broadly neutralizing across a wide panel of B and C type HIV-1 viruses, whereas Fab 8062 is non-neutralizing. The crystal structures reveal important differences in the conformations of the CDR-H2 loops in the complexes that propagate into other regions of the antigen-antibody interface, and suggest that both neutralization properties and affinity for the target can be attributed, at least in part, to the differences in the interactions of the CDR-H2 loops with the antigen. Furthermore, modeling of the complex of an N-HR trimer with three Fabs suggests that the CDR-H2 loop may be involved in close intermolecular contacts between neighboring antibody molecules, and that such contacts may hinder the formation of complexes between the N-HR trimer and more than one antibody molecule depending on the conformation of the bound CDR-H2 loop which is defined by its interactions with antigen. Comparison with the crystal structure of the complex of 5-Helix with another neutralizing monoclonal antibody known as D5, derived using an entirely different antibody library and panning procedure, reveals remarkable convergence in the optimal sequence and conformation of the CDR-H2 loop.

## Author Summary

Membrane fusion of HIV-1 with its target cells represents the first step in viral infection. This process involves a series of conformational changes in two viral envelope glycoproteins, gp120 and gp41, subsequent to binding of gp120 to the CD4 receptor and the chemokine coreceptor on the target cell membrane. During the fusion process, the conserved N-heptad repeat (N-HR) of gp41 in the form of a trimeric coiled-coil is accessible and presents an attractive target for the generation of broadly neutralizing antibodies. Here we present the crystal structures of two monoclonal Fabs complexed to a mimetic of the N-HR trimer. These Fabs were derived from a synthetic human combinatorial antibody library comprising more than 10^10^ human specificities by first panning against an N-HR mimetic, followed by affinity maturation through targeted diversification of the CDR-H2 complementarity determining region. One of the Fabs is broadly neutralizing across a wide range of primary isolates from subtype B and C HIV-1, whereas the other one is non-neutralizing. Our structures reveal the key role of the CDR-H2 loop in antigen recognition and how this correlates with HIV-1 neutralization properties.

## Introduction

The initial steps of fusion of HIV-1 virus to host cells involve binding of the HIV-1 surface envelope (Env) glycoprotein gp120 to the primary receptor CD4 and the chemokine co-receptor CXCR4 or CCR5 [1], [2]. These binding events trigger a series of conformational changes in both gp120 and the associated Env glycoprotein gp41 that lead to the formation of a so-called pre-hairpin intermediate (PHI) of the ectodomain of gp41 [3]. In the PHI, the C-heptad repeat (C-HR; residues 623–663) and the helical coiled-coil trimer of the N-heptad repeat (N-HR, residues 542–591) do not interact with one another, but rather bridge the viral and target cell membranes. The C-terminal transmembrane region of gp41 remains inserted into the viral membrane and the N-terminal fusion peptide of gp41 is inserted into the target cell membrane [3]–[5], [2], [6]. Subsequent apposition of the trimeric N-HR coiled-coil with three C-HR's results in the formation of a six-helix bundle (6-HB) that brings the viral and cell membranes into close proximity, eventually leading to their fusion [7]–[10]. The PHI constitutes an attractive target site for fusion inhibitors since both the N-HR and C-HR are accessible [11]–[31]. Moreover, the N-HR is highly conserved across a wide range of HIV-1 strains, and it has recently been shown that neutralizing antisera can be elicited by vaccination with a disulfide stabilized, trimeric peptide mimetic of the N-HR [32].

Recently, a number of monoclonal antibodies directed against the N-HR of gp41, many of them shown to neutralize HIV-1 to varying degrees, have been reported [33]–[40]. One such antibody, D5 [34], [41], was derived from a naïve human scFv library selected by panning against an inner core mimetic of gp41, known as 5-Helix. The 5-Helix construct comprises a single chain in which the N-HR trimeric coiled-coil is surrounded by only two C-HR helices, thereby exposing one face (comprising two N-HR helices) of the internal trimeric N-HR coiled-coil [15]. A crystal structure of D5 complexed to 5-Helix (PDB code 2CMR) reveals that one of the predominant interactions involves the complementarity determining region CDR-H2 loop of D5 protruding into the conserved hydrophobic pocket of 5-Helix [41].

In previous studies [35], [39] we reported a series of broadly neutralizing mini-antibodies derived from a synthetic human combinatorial antibody library (HuCAL GOLD [42]), comprising more than 10^10^ human specificities, by panning against the chimeric gp41-derived construct N_CCG_-gp41 [16]. The latter molecule exposes, in a stable manner, the complete N-HR internal trimeric coiled coil in the form of a disulfide-linked trimer. The parental Fab 3674 [35] was subjected to affinity maturation against the N_CCG_-gp41 antigen using targeted diversification of the CDR-H2 loop which resulted in significant enhancement of HIV-1 neutralization properties, both in terms of IC_50_ and neutralization breadth, over a standard panel of Envs from contemporary primary isolates of HIV-1 subtypes B and C [39]. Indeed, the best affinity-matured Fab in a monovalent format was comparable in its neutralization potency to the parental Fab 3674 in a bivalent format. The structural basis of such properties was, however, not previously characterized. Here we report the crystal structures of two affinity matured Fabs from this series (8066 and 8062), each complexed to 5-Helix. These Fabs represent the extremes of this series, since Fab 8066 was the most potent of the affinity matured Fabs, whereas Fab 8062 did not exhibit any detectable neutralization activity. The affinity of both Fabs for N_CCG_-gp41, determined by solution equilibrium titration using an electrochemiluminescence-based affinity measurement, was comparable (*K_D_* ∼15 nM) [39], although the affinity of Fab 8066 for 5-Helix measured by isothermal calorimetry (this work) is more than two orders of magnitude higher than that of Fab 8062. The crystal structures reveal important differences in the conformations of the CDR-H2 loops of the Fabs in the two complexes that provide a structural basis for the differences in neutralization properties and affinity for 5-Helix of the two Fabs.

## Results

### Characterization of Fab 8062 and 8066 complexes with 5-Helix

**Figure 1:**
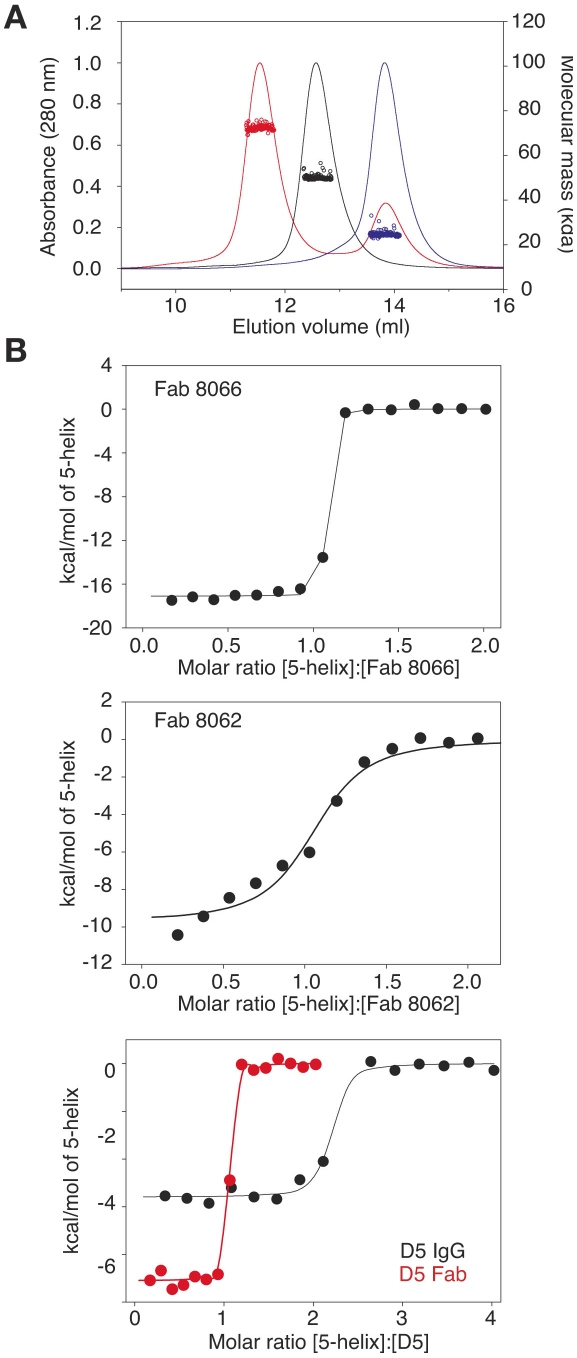
Characterization of the interaction of Fab 8066, Fab 8062 and the D5 antibody with 5-Helix. (A) Molecular mass analysis of 5-Helix (blue), Fab 8066 (black) and a mixture of Fab 8066 and 5-Helix in a 1∶2 molar ratio (red) by analytical size-exclusion chromatography with inline multiangle light scattering and refractive index measurements (SMR). Measured average masses are 72440±1014 Da for the Fab 8066/5-Helix complex, 49480±594 Da for Fab 8066 (calculated 48,896 Da) and 24680±592 Da for 5-Helix (calculated 24,459 Da). (B) ITC measurements for the binding of Fab 8066 (top), Fab 8062 (middle) and the D5 antibody (bottom) to 5-Helix in 10 mM Tris buffer, pH 7.5, 150 mM NaCl at 28°C. Thermal changes were monitored on addition of 2.45-µl aliquots of 100–150 µM 5-Helix solution to 5–15 µM Fab/antibody in the calorimetric cell (volume 0.2049 ml). Binding constants and thermodynamic parameters obtained by curve fitting of the integrated data to a simple single site binding model are provided in Table 1.

To assess complex formation between the Fabs and 5-Helix, we carried out size exclusion chromatography with detection by multi-angle light scattering and refractive index (SMR) (Fig.1). Stable complex formation was demonstrated by the appearance of a single peak that is retained with a mass of 72440±1014 Da (Fig. 1A, red). The peaks corresponding to Fab 8066 (Fig. 1A, black) and 5-Helix (Fig 1A, red) have masses of 49480±594 and 24680±592, respectively. The calculated masses of Fab 8066 and 5-Helix are 48896 and 24459, respectively.

The affinity for 5-Helix of Fab 8066 and 8062, as well as the D5 antibody, was assessed by isothermal titration calorimetry (Fig. 1B and Table 1). The stoichiometry of binding determined by ITC is 1∶1 for the complexes of the two Fabs (8066 and 8062) with 5-Helix, in agreement with the SMR data. The D5 monoclonal antibody, however, is a bivalent full-length IgG, and hence the stoichiometry of 5-Helix to D5 is 2∶1. The *K_D_* values for the binding of Fab 8066, D5 (Fab and IgG) and Fab 8062 to 5-Helix are <<10 nM, 10–20 nM and ∼200 nM, respectively. For these three antibodies, the binding data with 5-Helix correlate with neutralization activity. The HIV-1 neutralization potency of Fab 8066 (in monovalent format) is 1.5 to 4-fold higher than that of both the D5 IgG and Fab in an Env-pseudotyped neutralization assay using Envs from four laboratory-adapted strains of HIV-1 (HXB2, SF162 JR CSF, and 89.6). Fab 8062, on the other hand, displays no neutralization activity towards these four strains (Table 1). It should also be noted that the bivalent format of Fab 8066 is 2-7 fold more potent than the monovalent format in terms of neutralization activity over a large panel of subtype B and C HIV-1 strains [39].

**Table 1:** Thermodynamics of 5-Helix binding and HIV-1 neutralization activity.

Antibody	Binding to 5-Helix	HIV-1 neutralization IC_50_ (nM)^b^			
	*K_D_* (nM)^a^	HXB2	SF162	JR CSF	89.6
Fab 8066	<<10	85±7	550±80	490±50	550±70
Fab 8062	∼200	NA^c^	NA^c^	NA^c^	NA^c^
Fab D5	∼10	300±40	1300±300	640±140	890±200
IgG D5	<20	230±40	940±160	640±80	2240±350

a ITC measurements were carried out at 28°C in 10 mM Tris buffer, pH 7.5, 150 mM NaCl.

b HIV-1 neutralization activity was measured using an Env-pseudotyped virus neutralization assay as described previously [39] with Envs from four HIV-1 subtype B laboratory-adapted strains, HXB2, SF162, JR CSF and 89.6. Fabs 8066 and 8062 are in monovalent format.

c NA, no activity. Neutralization was too weak to reliably determine an IC_50_.

### Overall characteristics of the antibody-binding epitope on 5-Helix

**Figure 2:**
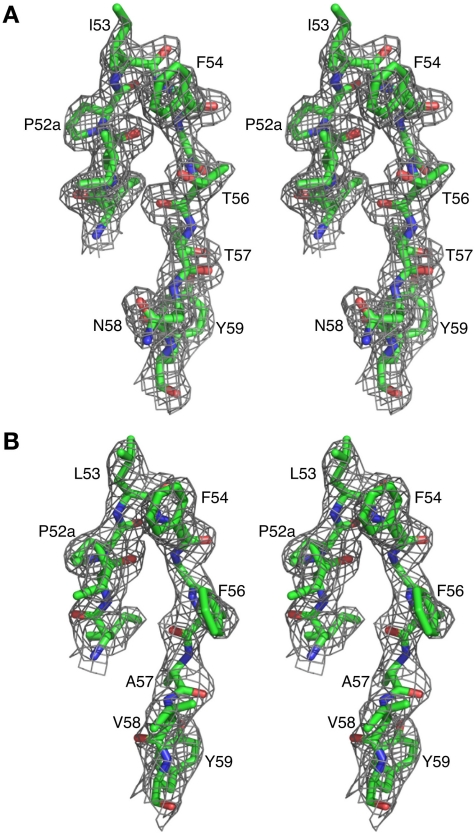
Representative *2F_o_*-*F_c_* electron density maps. CDR-H2 loop (residues 51–59) in (A) the Fab 8066/5-Helix complex and (B) the Fab 8062/5-Helix complex contoured at 1.5 and 1.0 σ, respectively.

The crystal structures of Fab 8066 and 8062 complexed to 5-Helix were determined at 2.05 and 2.5 Å resolution, respectively (Table 2). Representative electron density maps for the CDR-H2 loops of both complexes are shown in Fig. 2. The structures of these two Fabs were compared to the previously published 2 Å resolution crystal structure of a complex of the D5 antibody with 5-Helix [41]. The 5-Helix construct can be denoted as *Na*-*L*-*Ca*-*L*-*Nb*-*L*-*Cb*-*L*-*Nc*, where *Na*, *Nb* and *Nc* are the three N-HR helices that form the internal trimeric coiled-coil of gp41, *Ca* and *Cb* are the first two C-HR helices of the 6-HB of gp41, and the *L* segments are 5-residue linkers; the *Cc* helix of the 6-HB is absent in 5-Helix [15]. In the context of the 6-HB trimer, *Na* and *Ca*, *Nb* and *Cb*, and *Nc* and *Cc* belong to three separate subunits. It should be noted that identification of the helices is different here than in the D5 complex, where the numbering of residues was incorrect by not depicting the actual connectivity of helices in 5-Helix (for details, see Figure S1 in Supporting Information S1). Since the amino acid sequences are identical among the three *N* helices, as well as in the two *C* helices, such a numbering change does not affect the nature of the intermolecular interactions between the Fabs and 5-Helix. However, reinterpretation of the connectivity of helices does change the assignment of intra- and intersubunit interactions when interpreted in the context of the 6-HB trimer.

**Table 2:** Crystallographic data collection and refinement statistics.

	Fab 8066/5-Helix	Fab 8062/5-Helix
**Data collection**		
Wavelength (Å)	1.000	1.000
Space group	*P*2_1_2_1_2_1_	*P*2_1_
Unit cell parameters (Å)	*a* = 41.5, *b* = 124.7, *c* = 133.7	*a* = 83.7, *b* = 41.7, *c* = 98.6
		β = 94.85°
Resolution (Å)	50.0–2.04	50.0–2.5
Number of reflections (unique/total)	44,497 (213,954)	23,655 (73,516)
Completeness (last shell)	99.2% (96.6%)	99.1% (96.7%)
*R_merge_*	9.9% (66.2%)	8.2% (52.4%)
**Refinement**		
No. of complexes in a.u.	1	1
No. of protein atoms	4794	4805
No. of solvent molecules	443	69
No. of heteroatoms	2	0
*R_cryst_*	19.3%	20.0%
*R_free_* (3%)	24.5%	26.4%
r.m.s. deviation from ideality		
Bond lengths (Å)	0.010	0.010
Bond angles (°)	1.21	1.19
Ramachandran		
most favored (%)	94.4	92.4
additionally allowed (%)	5.6	7.6
PDB ID	3MA9	3MAC

**Figure 3:**
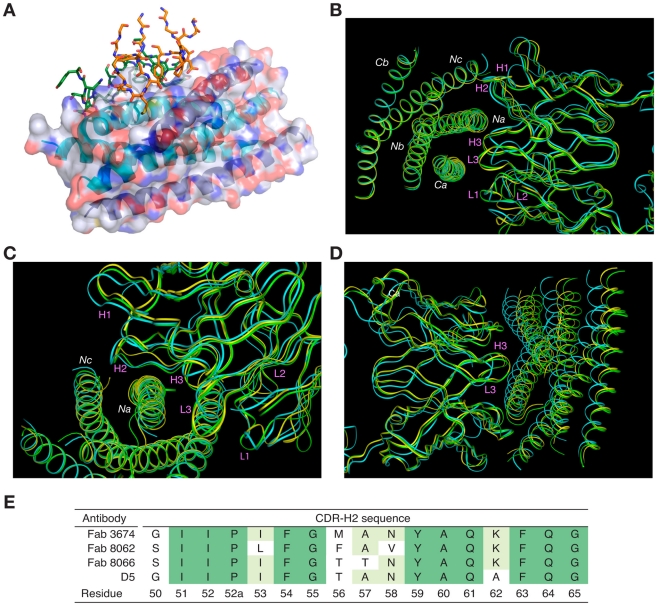
Overall view of the Fab/5-Helix complexes. (A) Interaction of all the CDRs with 5-Helix in the Fab 8066 and Fab 8062 complexes. The CDRs are shown as stick models with the carbon atoms in green for Fab 8066 and in orange for Fab 8062. 5-Helix is shown as a ribbon with the N-helices in cyan and the C-helices in dark blue. A transparent molecular surface of 5-Helix is also shown with the accessible surfaces of positively and negatively charged residues depicted in blue and red, respectively. (B-D) Superposition of the backbone of the Fab 8066, Fab 8062, and D5 complexes with 5-Helix, displayed as green, yellow and cyan tubes, respectively, superimposed on the Cα atoms of 5-Helix in (B) and (C) and on the Cα atoms of the antibodies in (D). (E) Comparison of the CDR-H2 sequence of the parental Fab 3674 with that of Fabs 8066 and 8062, as well as the D5 antibody [34]. The residue numbering of the CDR-H2 is according to [51].

The overlapping epitope, which is recognized by both Fabs on the surface of 5-Helix (Fig. 3A), is similar to the one described in the structure of the D5/5-Helix complex. This epitope comprises three helices, *Na*, *Nc* and *Ca*, out of the five present in 5-Helix (Figs. 2B,C). The *Na* helix is located in the middle of the epitope and thus contributes residues to the interactions with five CDRs of the antibodies. Two CDRs, H1 and H2, interact with residues in the groove located between helices *Nc* and *Na*, while the other two CDRs, H3 and L3, interact with residues in the groove located between helices *Na* and *Ca*. CDR-H2 covers the groove between *Na* and *Nc* and is positioned primarily over the *Na* helix (Figs. 3B,C).

The total accessible surface area buried at the antigen/antibody interface is comparable for the Fab 8066 and 8062 complexes with 5-Helix (∼2300 Å^2^) and divided approximately equally between antigen and antibody. This area is ∼250 Å^2^ greater than for the D5/5-Helix complex. The interactions with the heavy chain CDRs bury about twice as much accessible surface as those with the light chain CDR's. The number of residues at the interface (defined as residues whose accessible surface area decreases by >1 Å^2^ upon complexation [43]) is also similar for the Fab 8066 and 8062 complexes (∼60 residues subdivided equally between antibody and antigen) and somewhat larger than for the D5 complex (53 residues, 28 from the antibody and 25 from 5-Helix).

Despite the overall similarity of the three antibody or Fab complexes with 5-Helix, there are significant differences in the conformations of the corresponding CDRs, as well as in the conformation of the interacting residues within the antibody-antigen interface. These differences are strongly correlated with the variations in the sequences of the CDRs. The amino acid sequences of all CDRs other than CDR-H2 differ markedly between D5 and Fab 8066, leading to obvious structural differences in these segments. Fortuitously, the CDR-H2 of Fab 8066 and D5 converged to highly homologous sequences in the selection procedures used to generate these antibodies (Fig. 3E), leading to similarities in the conformation of this part of their structures. By way of contrast, the CDR-H2 sequences of Fabs 8066 and 8062 diverged during affinity maturation (Fig. 3E), resulting in significant differences in their conformations. These conformational differences, as well as the altered interaction patterns, may be reflected in different biological properties of these three antibodies, as manifested by the binding and neutralization data shown in Table 1.

The conformations of CDRs H1, H3, and L3 of Fabs 8066 and 8062 in the complexes with 5-Helix and their interactions with the antigen are most similar. CDRs H3 and L3 interact with residues from *Na* and *Ca* helices, and therefore are positioned in a shallow groove between these two helices on the surface of 6-HB (Fig. 3B). Detailed analysis of the interactions between the CDRs and the antigen (Table S1 in Supporting Information S1) indicates that, prior to affinity maturation of CDR-H2, CDR-L3 and CDR-H3 carried most of the interface interactions. The loops in both CDR-L3 and CRD-H3 are shorter in the D5 antibody (by 2 residues each), accounting for the rigid body movement of 5-Helix towards the antibody, as well as for the reduced number of the antigen-antibody interactions in the D5 complex, compared to the two Fab complexes. The effect of the differences in length of CDR-L3 and CDR-H3 is illustrated in Fig. 3D, where all three complexes are superimposed based on the Cα coordinates of the antibodies.

### Comparative analysis of the individual CDRs in the three complexes

**Figure 4:**
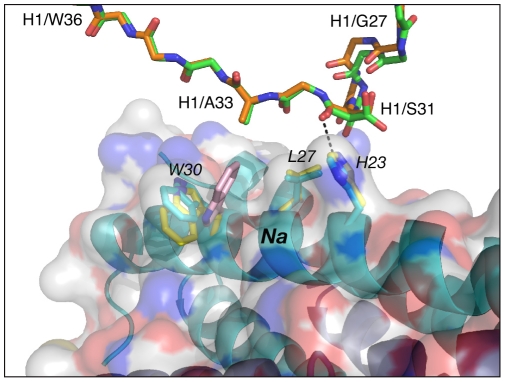
Comparison of the interactions of CDR-H1 of Fabs 8066 and 8062 with 5-Helix. The color coding is as follows: side chains of Fab 8066 and 8062, green and orange carbon atoms; side chains of 5-Helix in the Fab 8066 and 8062 complexes, cyan and yellow, respectively. The position of *Trp-30* of 5-Helix in the D5 complex [41] is shown in pink. A transparent molecular surface of 5-Helix is displayed with the accessible surfaces of positively and negatively charged residues colored in blue and red, respectively.

The conformation adopted by CDR-H1 in the complexes of Fabs 8066 and 8062 with 5-Helix is very similar. Two residues of CDR-H1, Ser-31 and Ala-33, interact with *His-23* and *Leu-27* of helix *Na* of 5-Helix, and their mode of interactions is essentially the same in both complexes (Fig. 4) (Italics are used throughout for residues of 5-Helix.) Although the side chain of Ser-31 is found in two orientations in the Fab 8066 complex, compared to a single conformation in the Fab 8062 complex, a long hydrogen bond between its carbonyl oxygen and the Nε2 atom of *His-23* is present in both complexes. The other contacts involving CDR-H1 are hydrophobic. When compared to the D5 complex, an additional hydrophobic contact is made between Ala-33 and *Trp-30*, due to the different orientation of the latter's side chain (Fig. 4 and Table S1 in Supporting Information S1).

**Figure 5:**
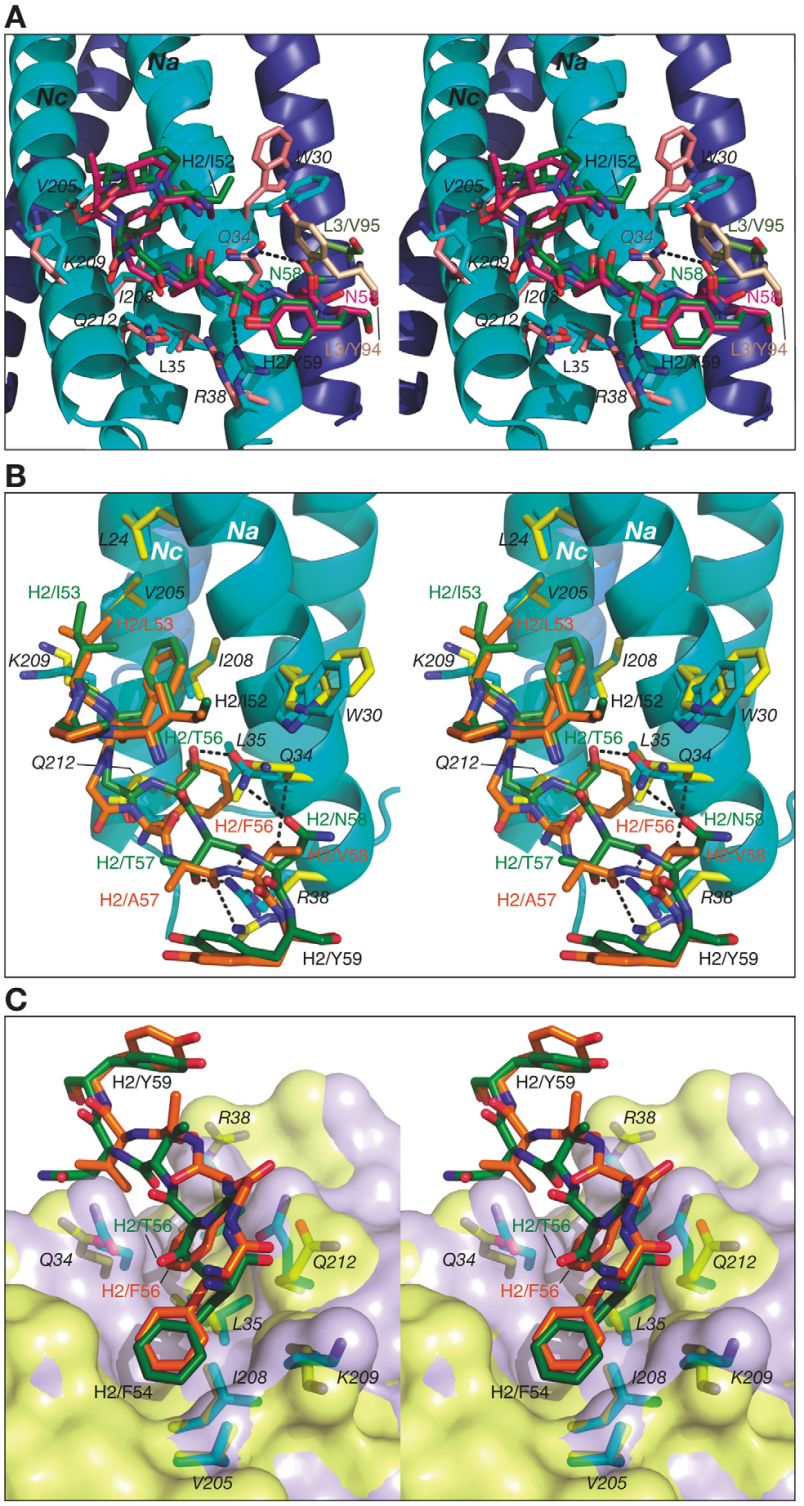
Stereoviews showing the interactions of CDR-H2 of Fab 8066, Fab 8062 and D5 with 5-Helix. (A) The Fab 8066 complex versus the D5 complex [41]. (B) and (C) The Fab 8066 complex versus the Fab 8062 complex. Color coding: the carbon atoms of side chains of Fab 8066, Fab 8062 and D5 are shown in green, orange and magenta, respectively; the carbon atoms of side chains of 5-Helix in these three complexes are shown in cyan, yellow and pink, respectively. Helices of 5-Helix are shown as ribbons with the N-helices in cyan and the C-helices in dark blue. A transparent molecular surface of 5-Helix is included in (C), colored in purple for the Fab 8066 complex and in yellow for the Fab 8062 complex.

Similarly to CDR-H1, CDR-H2 interacts with residues located in a groove between helices *Nc* and *Na* of 5-Helix (Fig. 3C). CDR-H2 in the antibody/5-Helix complexes effectively substitutes for the N-terminal end of the *Cc* helix that is present in the six-helix bundle of complete gp41, but is absent in 5-Helix. The significance of the interactions made by CDR-H2 residues might be attributed to the unique orientation of this loop, which lies parallel to the surface of the epitope and covers the groove like a lid (Fig. 3C). This mode of interaction distinguishes CDR-H2 from the other CDRs which are all oriented approximately perpendicular to the interface. Because of the unique position of CDR-H2, every residue of this loop is involved in the interactions with the antigen. The amino acid sequence of the CDR-H2 loop converged during selection to be almost identical in Fab 8066 and the D5 antibody, with the exception of a single residue (Thr-57 in Fab 8066 is an Ala in D5), and therefore the interactions of CDR-H2 with 5-Helix are very similar in both complexes (Fig. 5A). The interactions involving CDR-H2 are predominantly hydrophobic, and the few polar interactions that are present vary between the two complexes (Table S1 in Supporting Information S1). Two such polar interactions are unique to the Fab 8066 complex, and involve a short hydrogen bond between the side chains of Thr-57 of CDR-H2 and *Arg-38* of the *Na* helix, as well as a hydrogen bond between the side chains of Asn-58 and *Gln-34* (Fig. 5A). In the D5 complex, Asn-58 is forced to adopt a different conformation due to the close proximity of the bulky side chain of Tyr-94 of CDR-L3. The structural equivalent of the latter residue in Fab 8066 is the much smaller Val-95 (Fig. 5A).

In the case of Fabs 8066 and 8062, the sequence of CDR-H2 diverged during affinity maturation (Fig. 3E), leading to a different conformation of this loop, as well as to variations of the interaction pattern in the two complexes (Fig. 5B). Nevertheless, a majority of the hydrophobic interactions are preserved for those residues of CDR-H2 that are either identical or of a similar type (for example, Ile/Leu-53) in both Fabs. Some of the polar interactions in the Fab 8062 complex are lost, and compensated by additional hydrophobic interactions (Table S1 in Supporting Information S1). For example, a hydrogen bond between Asn-58 of CDR-H2 and *Gln-*34 of the *Na* helix, present in the Fab 8066 complex, is substituted by hydrophobic interactions between the side chain of Val-58 of Fab 8062 and the same residue of 5-Helix (*Gln-34*). However, the hydrogen bond between Thr-57 of CDR-H2 and *Arg-38* of the *Na* helix in the Fab 8066 complex has no equivalent in the Fab 8062 complex due to the Thr-57Ala substitution in the latter. A hydrogen bond between the carbonyl oxygen of Thr/Ala-57 and the side chain of *Arg-38* is present in both complexes (Fig. 5B). A hydrogen bond formed between the side chains of Thr-56 and *Gln-34* of the *Na* helix in Fab 8066 complex is not found in the Fab 8062 complex, since the latter has a Phe in the corresponding position. In turn, the large Phe side chain is involved in extensive hydrophobic interactions between Fab 8062 and 5-Helix. As a result, the number of contacts between the two Fabs and 5-Helix is comparable. Nevertheless, when the topography of the CDR-H2 loops within two Fab complexes is reviewed, a significant difference can be noticed (Fig. 5C). Substitution of Thr-56 in Fab 8066 by Phe-56 in Fab-8062 results in shifting of the whole segment of the CDR-H2 loop out of the groove on the surface of the bundle, where it was docked in the Fab 8066 complex. Thus, while the hydrophobic interactions between Phe-56 and 5-Helix are preserved, including an unfavorably short one (3.2 Å) with *Gln-212* of the *Nc* helix, this substitution results in displacement of the main chain surrounding Phe-56 away from 5-Helix.

**Figure 6:**
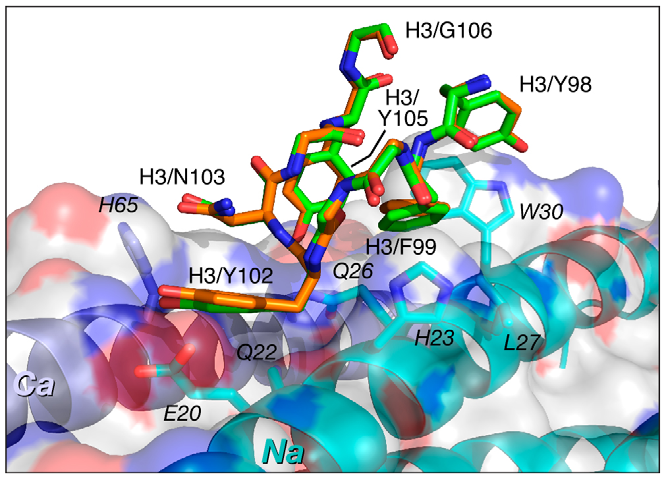
Comparison of the interactions of CDR-H3 of Fab 8066 and Fab 8062 with 5-Helix. Color coding: the carbon atoms of side chains of Fab 8066 and Fab 8062 are shown in green and orange, respectively. Only side chains of 5-Helix in the Fab 8066 complex are shown with carbon atoms in cyan (as they are essentially identical in the Fab 8062 complex). The *Na* and *Ca* helices of 5-Helix are shown as cyan and dark blue ribbons. A transparent molecular surface of 5-Helix is also included with the accessible surface of positively and negatively charged residues in blue and red, respectively.

The CDR-H3 sequences are identical in Fabs 8066 and 8062, and these loops maintain very similar conformations in the complexes with 5-Helix. As noted above, residues of CDR-H3 interact with the residues in the groove between the *Na* and *Ca* helices of 5-Helix, maintaining very similar contacts, with only a slight variation in the distances between the same pairs of atoms (Fig. 6). The interactions are predominantly of a hydrophobic nature, with only a few polar interactions. One strong hydrogen bond between the hydroxyl of Tyr-102 and *His-65* (2.7 Å) in the Fab 8066 complex is lost in the Fab 8062 complex, due to different orientation of the side chain of *His-65* in the two complexes. *His-65* has two alternate conformations in the Fab-8066 complex, but only a single one in the Fab 8062 complex, most likely due to the differences in the conformation of CDR-L1 in the two complexes. The hydrogen bond between Tyr-102 and *His-65* in the Fab 8066 complex is formed with the histidine side chain adopting the conformation that is absent in the Fab 8062 complex.

**Figure 7:**
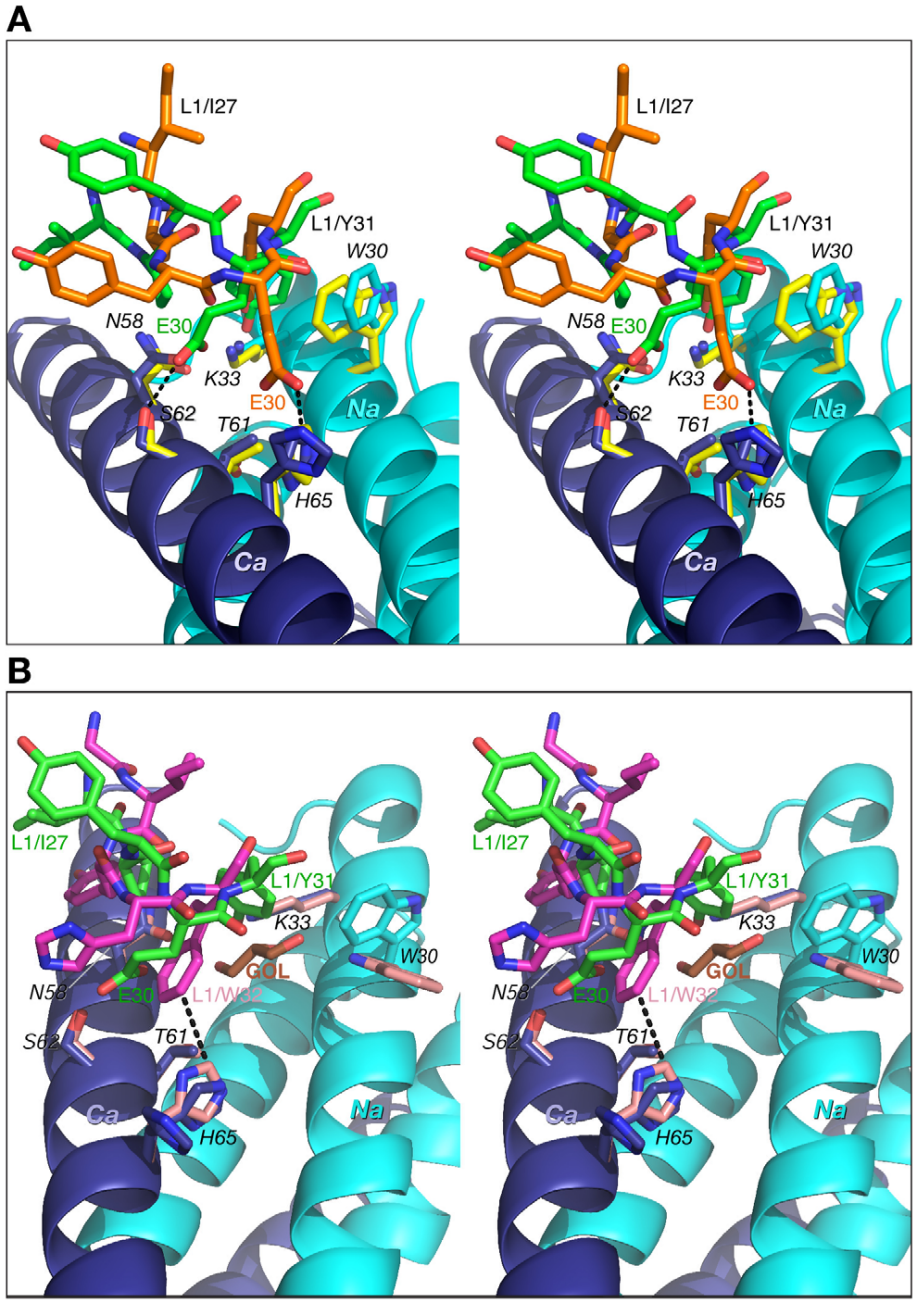
Comparisons of the interactions of CDR-L1 of Fab 8066, Fab 8062 and D5 with 5-Helix. (A) The Fab 8066 complex versus the Fab 8062 complex. (B) The Fab 8066 complex versus the D5 complex [41]. Color coding: the carbon atoms of side chains of Fab 8066, Fab 8062 and D5 are shown in green, orange and magenta, respectively; the carbon atoms of side chains of 5-Helix in the Fab 8066, Fab 8062 and D5 complexes are shown in cyan, yellow and pink, respectively, for the *Na* helix, and in blue, yellow and pink, respectively, for the *Ca* helix. The N- and C-helices of 5-Helix are shown as cyan and blue ribbons, respectively. The glycerol molecule (labeled GOL) in the D5 complex is shown in orange in (B).

The number of contacts involving interactions of CDR-L1 and CDR-L2 with 5-Helix is much lower than for the other CDRs discussed above. The conformation of CDR-L1 is different in each of the three antibody complexes compared here. However, as the CDR-L1 sequences are identical in Fabs 8066 and 8062, their interactions with 5-Helix are similar, with differences attributed to crystal contacts present in the Fab 8066 complex (Fig. 7A). CDR-L1 maintains the interactions on the periphery of the epitope, mostly with residues from helix *Ca* (Fig. 3B). Only two residues with bulky side chains, *Trp-30* and *Lys-33* from helix *Na*, extend to CRD-L1 and interact with the side chain of Tyr-31, and these intermolecular contacts are shorter in the Fab 8066 complex (Fig. 7A). The side chains of Glu-30 adopt different conformations in the two complexes. In the Fab 8066 complex Glu-30 is hydrogen bonded to *Ser-62*, whereas in the Fab 8062 complex this residue forms an ion pair with *His-65*. One additional hydrophobic interaction in the Fab 8066 complex that is not present in the Fab 8062 complex is found between Pro-28 and the Cγ atom of *Asn-58*; in the Fab 8062 complex Pro-28 is oriented away from the bundle. Although CDR-L1 in the D5 complex recognizes the same part of the 5-Helix epitope as the corresponding CDRs in the other two complexes, its conformation differs from both of them. Such different conformation of CDR-L1 in the D5 complex is due not only to the unique sequence of this loop, but also to the presence in the interface of a glycerol molecule from the cryoprotectant (Fig. 7B), which mediates antigen-antibody interactions [41].

**Figure 8:**
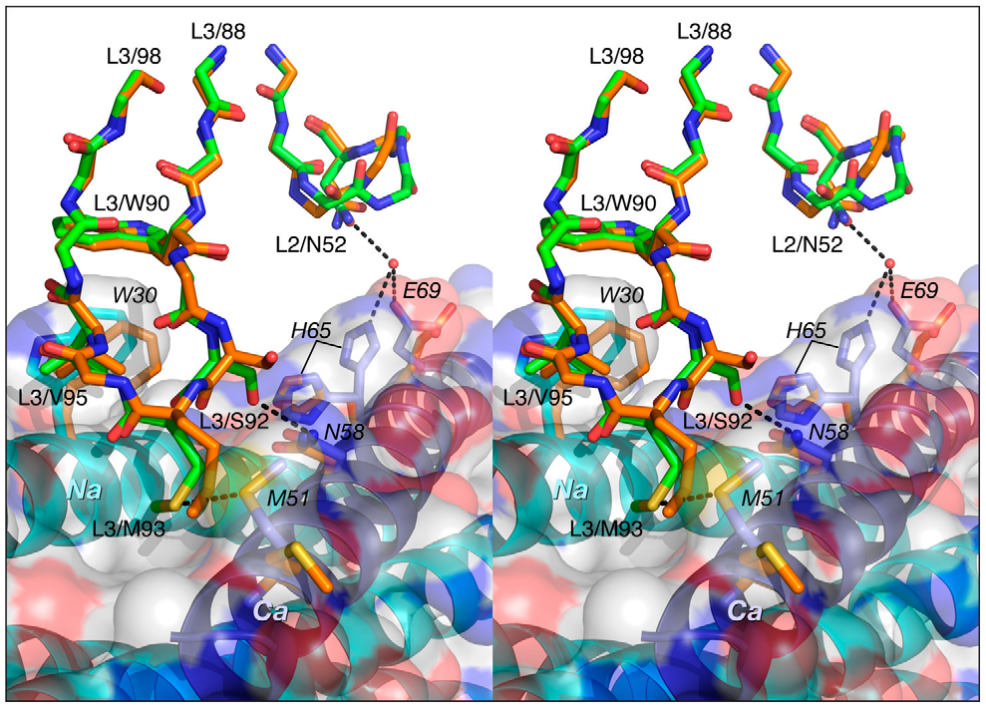
Comparison of the interactions of CDR-L2 of Fab 8066 and Fab 8062 with 5-Helix. Color coding: the carbon atoms of side chains of Fab 8066 and Fab 8062 are shown in green and orange, respectively; the carbon atoms of side chains of the *Na* and *Ca* helices in the Fab 8066 complex are in cyan and blue, respectively; the carbon atoms of the side chains of the *Na* and *Ca* helices in the Fab 8062 complex are in yellow. The *Na* and *Ca* helices of 5-Helix are shown as cyan and dark blue ribbons. A transparent molecular surface of 5-Helix is also included with the accessible surface of positively and negatively charged residues in blue and red, respectively.

The conformation of CDR-L2 is similar in all three complexes, except that in the Fab 8066 complex the tip of the CDR-L2 loop is oriented more towards 5-Helix then in the other two complexes. This allows formation of two solvent-mediated interactions with *His-65* and *Glu-*69 of helix *Ca* (Fig. 8). In the Fab 8062 complex, CDR-L2 is not involved in any contacts with the antigen.

The conformation of CDR-L3 is very similar in the Fab 8066 and 8062 complexes. The tip of this loop is oriented into the groove between helices *Na* and *Ca* (Fig. 8). In the Fab 8066 complex, two residues on the tip of CDR-L3, Ser-92 and Met-93, interact with two residues from the helix *Ca*, *Met-51* and *Asn-58*, respectively. No equivalent interactions are seen in the Fab 8062 complex, but several other hydrophobic contacts are similar in both complexes.

### Binding of CDR-H2 and CDR-H1 prevents the completion of the six-helix bundle

When the first 6-HB structures of the gp41 ectodomain emerged [7]–[10], key hydrophobic interactions between residues located on the N-terminus of a C-HR helix, and the C-termini of two adjacent N-HR helices in the trimer, were described. Several residues, such as *Trp-628*, *Trp-631* and *Ile-635* on the *C*-HR helix and *Ile-573* and *Val-570* (*Ile-208* and *Val-205* in the Fab 8066 and 8062 complexes, respectively) on the *N*-HR helix from the same molecule of gp41, maintain numerous intramolecular hydrophobic interactions (numbering for the *Cc* residues is taken from the 1AIK structure [7] and corresponds to the numbering of the native gp160 sequence). These interactions were identified to play a crucial role for the stability of the 6-HB and hence for successful initiation and completion of the fusion process [7]–[10].

**Figure 9:**
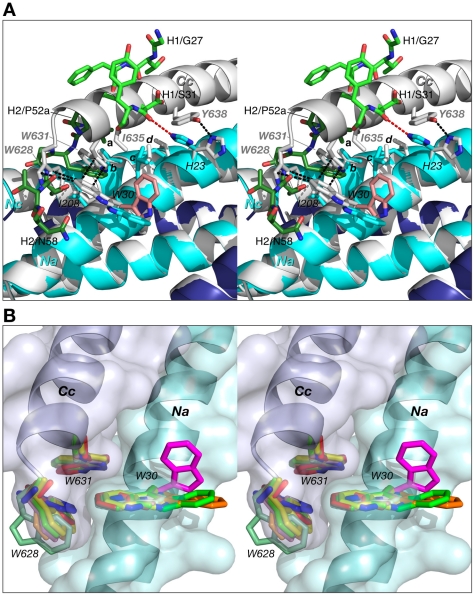
Comparison of 5-helix complexed to Fab 8066 with the 6-HB structure of gp41. (A) The 5-Helix bundle from the complex with Fab 8066 is superimposed onto the 6-HB trimer (both shown in cartoon representation, with the N-HR and C-HR helices in the Fab 8066 complex colored cyan and dark blue, respectively, and in white for 6-HB). Hydrophobic residues in contact within the interface between the third C-HR helix (denoted as *Cc*) that is absent in 5-Helix and the two adjacent N-HR helices (*Na* and *Nc*) are shown in sticks in the same color scheme as the cartoons. Selected side chains are labeled, with letters *a-d* denoting Ile-53 of the Fab CDR-H2, *Val-205* of helix *Nc* (*V570* in the 6-HB), *Leu-27* of helix *Na* (*L568* in the 6-HB), and *Leu-24* of helix *Na* (*L565* in the 6-HB), respectively. The side chain of *Trp-30* in the D5 complex is in pink. The interactions within the 6-HB are shown as black dashed lines. The CDRs H2 and H1 are shown as sticks, in dark and light green, respectively. The hydrogen bond between the carbonyl of Ser-31 of the CDR-H1 and the imidazole ring of His-23 in the Fab 8066 complex is marked by a red dashed line. (B) Structures of the intersubunit “tryptophan lock” in the 6-HB and 5-Helix. Nine pairs of interacting N-HR and C-HR helices from different molecules of gp41 are superimposed. Three sets of tryptophan side chains seen in 6-HB gp41 trimers, two X-ray structure, pdb codes 1AIK [7] and 1ENV [8], and one NMR structure, pdb code 1QCE [10], [52], are shown in white, blue and red sticks, respectively. Three complexes of 5-Helix with antibodies provide six independently determined structures of the “tryptophan lock”. Two sets of tryptophan residues from the complexes with Fabs 8066 and 8062, and the D5 antibody are shown in two shades of light green, yellow, and pink, respectively. The side chains of Trp-30 from the first N-HR helix (*Na*) in the three complexes, which are involved in interactions with the CDR-H2 of the antibodies, as opposed to the third C-HR helix (*Cc*) which is absent in 5-Helix, are shown in dark green, orange and magenta, respectively. N-HR and C-HR helices are shown in cartoon representation with the molecular surface colored in light cyan and blue, respectively.

When complexed to 5-Helix, the CDR-H2 and CDR-H1 of Fabs 8066 and 8062, as well as D5, occupy the space taken by N-terminal end of the C-HR helix (*Cc*) in the 6-HB structure. Indeed, the segment of the CDR-H2 in Fab 8066 comprising residues 51–58 literally takes the place of the position of the N-terminus of the third *C*-HR helix in the gp41 trimer. In particular, the side chains of Phe-54, Ile-53 and Thr-56 of CDR-H2 preserve the interactions with *Ile-*208 and *Val-205* of the *Nc* helix, substituting for the intrasubunit interactions between the *Nc* and *Cc* helices of the 6-HB (Fig. 9A). Interestingly, the conformations of the hydrophobic residues on the *Nc* and *Na* helices are not significantly affected by the binding of the antibody, and the topography of the surface between the two adjacent N-HR helices, *Nc* and *Na*, required for successful docking of the *Cc* helix seems to be unperturbed with only two exceptions, namely the side chains of *His-23* and *Trp-30* (*His-564* and *Trp-571* in gp41).

In the case of *His-23*, both the χ_1_ and χ_2_ angles adopt different rotamers, with the result that the hydrogen bond between the Nδ atom of *His-23* of the *Na* helix and the hydroxyl of *Tyr-638* of the *Cc* helix in the 6-HB is replaced by a hydrogen bond between the Nε atom of *His-23* and the carbonyl of Ser-31 of CDR-H1 in the Fab 8066/5-Helix complex (Fig. 9A). Likewise, the side chain of *Trp-30* in the Fab/antibody complexes with 5-Helix also changes its conformation relative to that found in the 6-HB: for the Fab 8066 and 8062 complexes there is a ∼180° flip in the χ_2_ angle, while for the D5 complex both the χ_1_ and χ_2_ rotamers are altered.

In the absence of Fab/antibody the intersubunit hydrophobic interactions between *Trp-30* of *Na* (*Trp-571* of a N-HR helix of gp41) and two tryptophans (*Trp-628* and *Trp-631*) of a C-HR helix are strictly conserved within all 6-HB and 5-Helix structures solved to date (Fig. 9B). (In 5-Helix these interactions occur between helices *Nb* and *Ca* and between *Nc* and *Cb*). It seems reasonable to suggest that the formation of such an intersubunit “tryptophan lock” serves to optimally align the hydrophobic moieties on a given C-HR helix and two adjacent N-HR helices to form a hydrophobic zipper along the axis of the bundle. In the context of HIV-1 neutralization, binding of Fabs 8066 and 8062 and the D5 antibody compete with the formation of the intersubunit “tryptophan lock”. Hence, even subtle differences in the stability of the complexes with various antibodies under conditions of ongoing competition can determine the success in blocking productive 6-Helix bundle formation vital for viral fusion.

### Modeling the complexes of the gp41 N-HR trimer with FAbs 8066 and 8062

The crystal structures of the Fab complexes with 5-Helix presented here involve a single antibody molecule substituting for one C-HR helix. Apposition of the viral and target membranes requires the conversion of the PHP in which the N-HR trimer is exposed to a 6-HB in which the C-HR helices are bound to the outside of the N-HR trimer. Success in the competition between antibody and C-HR helices for binding to the exposed N-HR trimer of the PHP may depend on the ability of the antibody to form a complex with more than one antibody molecule bound. In principle, up to three antibodies could bind to three equivalent sites on the N-HR trimer. Indeed, maximum antagonism between Fab 8066 and the N-HR trimer mimetic N_CCG_-gp41 in the HIV-1 neutralization assay is observed at a molar ratio of 3∶1 [39]: at this ratio the concentrations of free N_CCG_-gp41 (which has 3 Fab binding sites) available to bind the C-HR of gp41 and free Fab 8066 available to bind the N-HR trimer of gp41 are at a minimum. To evaluate the impact of the observed structural differences in the interactions of the CDR-H2′s of the different Fabs with 5-Helix, we modeled complexes of the three Fabs bound to a N-HR trimer.

Modeling is complicated by the fact that whereas the gp41 trimer is formed from three identical N-HR helices, only two of the three N-HR helices, *Na* and *Nc*, interact with the Fab in the crystal structures of the complexes with 5-Helix. As a result, the symmetry of the N-HR trimer is broken in the crystal structures. To generate models of the N-HR trimer with three Fabs we used as reference structures two coordinate sets comprising three N-HR helices (*Na*, *Nb*, and *Nc*) and the Fab, which were extracted from the two crystal structures of 5-Helix bound to single Fab molecule, 8066 or 8062. The fragment of the each reference structure, containing helices *Na* and *Nc*, that interact with the Fab in the 5-Helix complex, plus the Fab molecule itself, was treated as a rigid body unit to create a model of a trimer with three Fabs bound.

**Figure 10:**
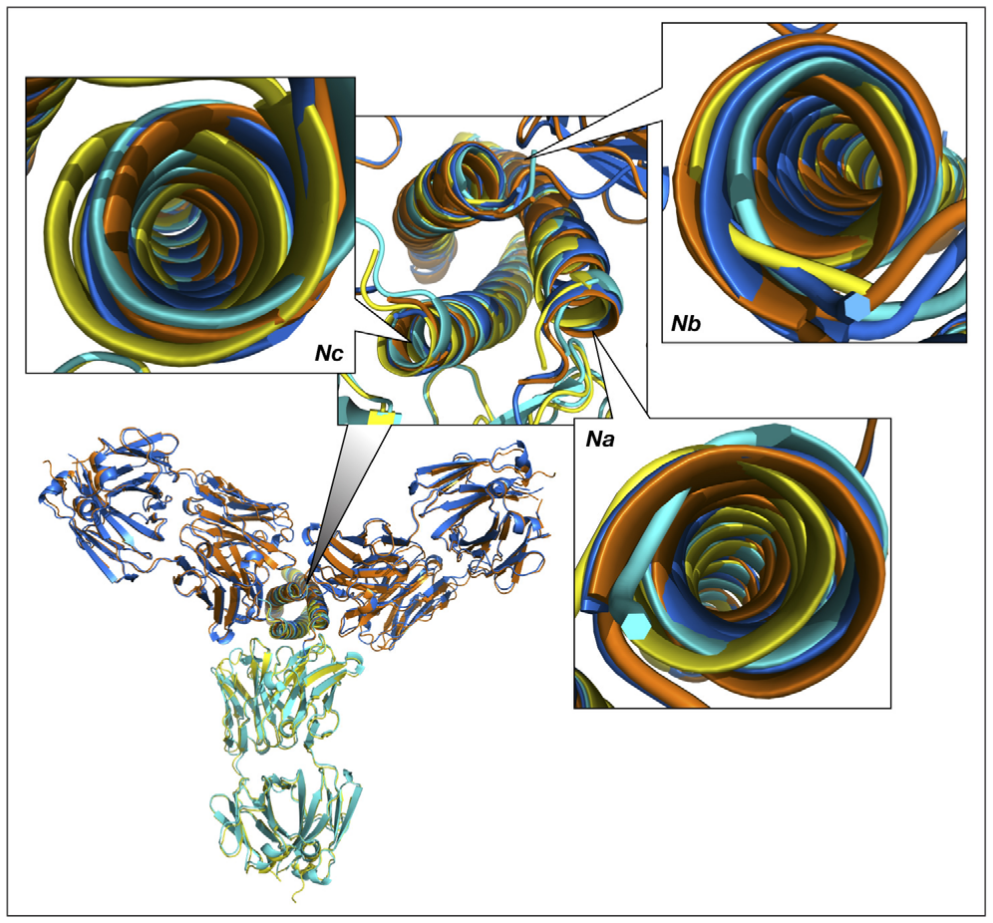
Superimposed models of the complexes of the trimer of N-HR helices of gp41 with three Fab molecules, built by sequentially superimposing two pairs of helices. The overall view of the models is shown in a cartoon representation. The crystal structures of the Fab complexes are colored cyan for Fab 8066 and yellow for Fab 8062, and the two modeled structures are colored navy blue and orange, respectively. The panels marked *Na*, *Nb* and *Nc* show the shifts between the experimental and modeled positions of the N-HR helices, using the same coloring scheme. Panel *Nb* shows two sets of helices in navy blue and orange, corresponding to their positions in the complexes with the Fabs positioned on each side of helix *Nb*.

We initially modeled the binding of two additional Fab molecules by superimposing two N-HR helices of the rigid body unit (*Na* and *Nc*) with two other helices of the trimer (first, with *Nc* and *Nb*, and, subsequently with *Nb* and *Na*). The aim of this approach was to preserve the intrinsic structure of the experimentally observed gp41 trimer. The results are shown in Fig. 10. The two modeled Fab 8066 molecules are reasonably well positioned in a pseudo-symmetric fashion between pairs of N-HR helices in the trimer, while two areas of steric clashes are observed for Fab 8062. The first involves slightly short contacts between CDR-L3 of one Fab and the CDR-H2 of another (2.2 Å between the side chain of Met-93 and the carbonyl oxygen of Phe-54, respectively). The second area displays short contacts for the interactions between CDR-H2 and helix *Nb* of the trimer (1.7 Å between the side chains of CDR-H2 Phe-54 and *Gln-124* of *Nb*). A closer look at the results of the modeling based on the superposition of two pairs of helices explains the latter as follows.

The three N-HR helices are involved in very different types of interactions in the 5-Helix complexes. The *Nb* helix interacts solely with C-HR helices on both sides, while the *Na* and *Nc* helices each interact on one side with a C-HR helix and on the other side with a Fab molecule. When the *Na* and *Nc* helices of the rigid body unit are superimposed on two other pairs of helices from a trimer, a shift in the resulting positions of that pair of helices is observed in the modeled structure, compared to their position in the original structure of the 5-Helix complex. This shift originates from the changes in the environment of each helix in the model, since all three N-HR helices are involved in a similar type of interactions with two Fabs on both sides. However, since helix *Nb* was used twice during model building to create two Fabs on either side of it, it has two distinct positions in the modeled structure, as can be clearly seen in Fig.10, panel *Nb*. Thus, to accommodate a Fab on the left side of helix *Nb*, helix *Nb* has to move to the right compared to its position in the gp41 trimer, and vice versa. The ambiguity of the positioning of helix *Nb* in the model demonstrates the shortcomings of this approach. We conclude that to build a model with three Fabs we should not constrain the structure of the helical trimer. Nevertheless, results of such modeling consistently indicate that the shifts in all three helices are larger when bound to Fab 8062 than to Fab 8066, suggesting that that formation of the complex with three Fab 8062 molecules requires more extensive structural rearrangements.

To address problems with the first model, we used an alternative approach in which only one helix out of two comprising the rigid body unit is used for sequential superpositions. In this manner, the second helix will be placed where it falls, and for the next round its new position will be used in the superposition for the purpose of creating the second modeled Fab. We fully realize the imperfections of such an approach, which will allow the trimer structure to relax without any restrictions applied. The advantage, however, is that the positions of each pair of helices is consistent with the presence of the Fab molecule bound between them. The resulting models are then only used for comparisons, although they are not considered to be accurate in absolute terms.

**Figure 11:**
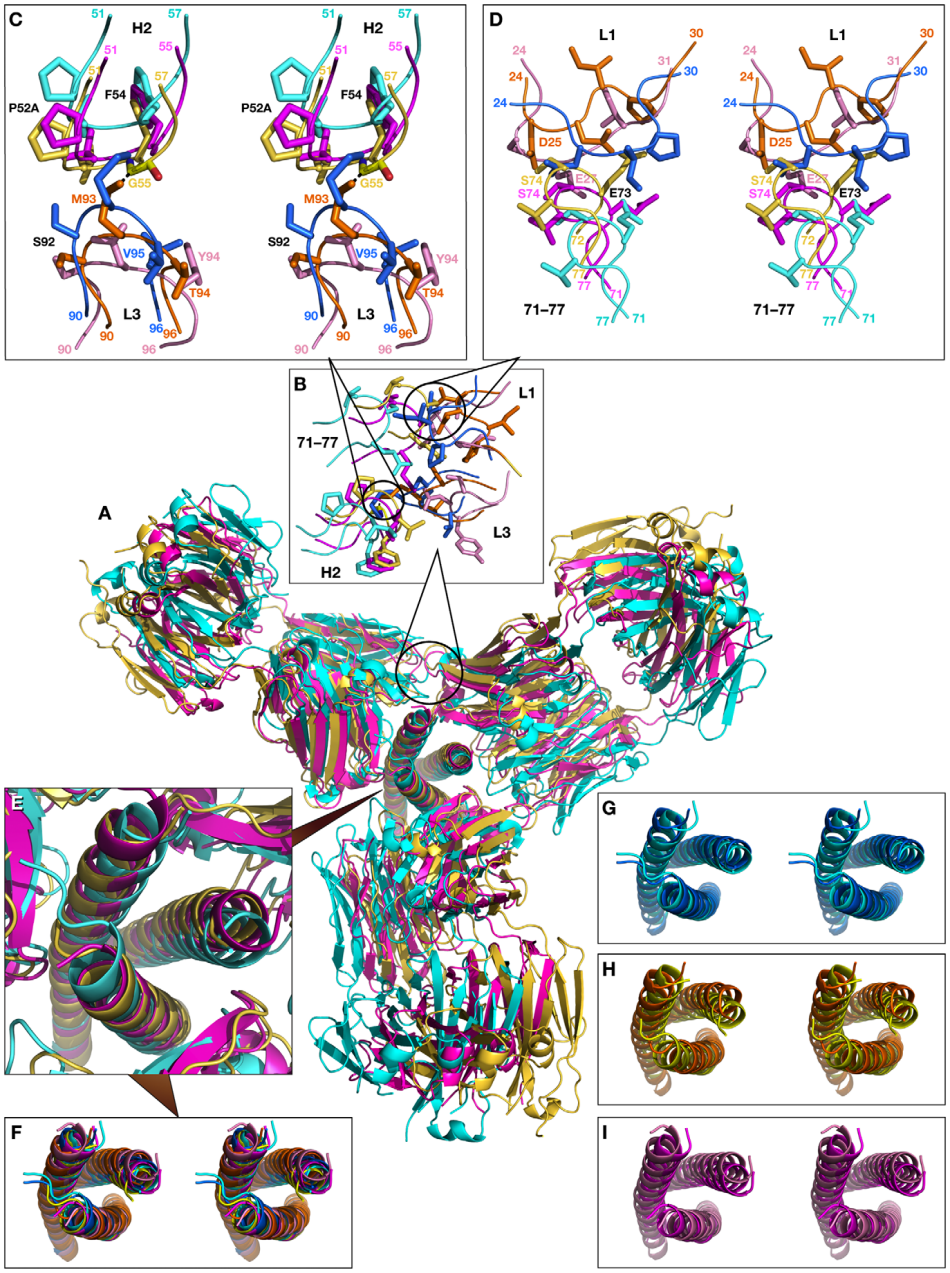
Superimposed models of the complexes of the trimer of N-HR helices of gp41 with three Fab molecules, built by sequentially superimposing single helices. (A) Overall view of the model shown in a cartoon representation. The modeled structures of the complexes are colored cyan for Fab 8066, yellow for Fab 8062, and magenta for D5. (B) Expansion of the area of steric clashes between neighboring Fab molecules. (C) Detailed view of the clashes between the CDR-H2 of one Fab and the CDR-L3 of another. (D) Detailed view of the steric clashes between the residues from the loop 71–77 of one Fab and the CDR-L1 of another. (E) Enlarged view of the trimer of N-HR helices in the models. (F) Stereoview corresponding to panel (E). (G), (H) and (I) Stereoviews of the superimposed trimers in crystal structures (light shades) and the models (dark shades) for Fab 8066, Fab 8062, and D5, respectively.

Fig. 11A shows the superposition of models of N-HR trimer-(Fab)_3_ complexes with Fabs 8066, 8062, and D5. Since we started to build the models for each Fab from the superimposed crystal structures, for clarity they are not shown. Similarly to the first model, the shift in the position of the third Fab is most pronounced for Fab 8062, less so for D5, and least for Fab 8066. This reflects the extent of rearrangements in the trimer upon increasing the ratio of Fabs to gp41 in the complexes. This is also confirmed by comparing the shifts of the helices for individual Fab complexes between the original and modeled structures (Fig. 11 G, H, I). The similarities of the first and the second models are extended to the areas with steric clashes at the interface between neighboring Fabs (Fig. 11B). The first area of steric clash involves the CDR-H2 of one Fab and the CDR-L3 of another (Fig. 11C), and the second clash area involves loop 71-77 of one Fab and the CDR-L1 of the other (Fig. 11D). Close contacts are only observed in the first area for Fab 8062 (between Gly-55 and Met-93, Fig 11C), whereas no bad intermolecular contacts are present for Fab 8066, owing to the different conformation of the CDR-H2. Since CRD-L3 is shorter in D5 than in the two others Fabs, it is also not involved in any collisions. Interestingly, comparison with the neutralizing antibodies DN9 (from a human B-cell library from an HIV infected patient, selected using the engineered disulfide stabilized N-HR trimer, N35_CCG_-N13), and 8K8 (raised in rabbits using N35_CCG_-N13) reveals a high level of sequence similarity of their CDR-H2′s with that of Fab 8066 [38]. In DN9, a residue with a longer side chain (Glu) occupies the position of Gly-55 (Table S2 in Supporting Information S1), but position 56 is occupied, as in Fab 8066, by Thr, which should allow avoidance of the collision described above. In contrast, position 56 in 8K8 is occupied by a long Arg side chain, but position 55 is still a Gly.

In the second area, short contacts are present in the trimer models of both Fab 8062 and D5, involving Ser-74 in both, as well as Asp-25 and Glu-27 of CDR-L1 in Fab 8062 and D5, respectively (Fig. 11D). The contacts between the neighboring Fabs in the model of the N-HR trimer-(Fab 8066)_3_ complex seem to be much better. There are no bad contacts between the Fabs and the individual helices in the N-HR trimer of the second model, because the positions of the helices were shifting as the model was being built to accommodate the Fabs.

## Discussion

Although Fab 8066 and the D5 monoclonal antibody were developed using completely different naïve antibody libraries with different panning and selection procedures [34], [35], [39], the interactions between their CDR-H2 loops and the hydrophobic pocket of 5-Helix converged to similar solutions (Figs. 2E and 5A). Affinity maturation of the CDR-H2 conveyed significant improvements in neutralization breadth and potency of Fab 8066 relative to its parental Fab 3674 [39], indicating that interactions with this region of gp41 are crucial for efficient neutralization of HIV-1.

Examination of the CDR-H2 sequences of a series of 10 affinity matured Fabs generated from the parental Fab 3674 [39], as well as the CDR-H2 sequences of D5 [41], DN9 and 8K8 [38] reveal that the key determinants relating to neutralization activity are correlated with the nature of the residues at positions 53, 54 and 56. In our series of affinity matured Fabs, 3 Fabs were broadly neutralizing (Fabs 8060, 8066 and 8068), 3 Fabs (8059, 8064 and 8069) as well as the parental Fab (3674) displayed weak neutralizing activity, and 4 Fabs (8061, 8062, 8063 and 8065) had no detectable neutralization activity. The neutralizing Fabs were all characterized by a Leu or Ile at position 53 with the exception of Fab 8064 which had a Trp; a Phe at position 54; and a polar residue at position 56. The presence of some of these key residues in CDR-H2 sequences, when reviewed in the framework of structural and modeling data presented in this study, seem to correlate with their affinity and biological activity, as shown by a few examples below.

**Figure 12:**
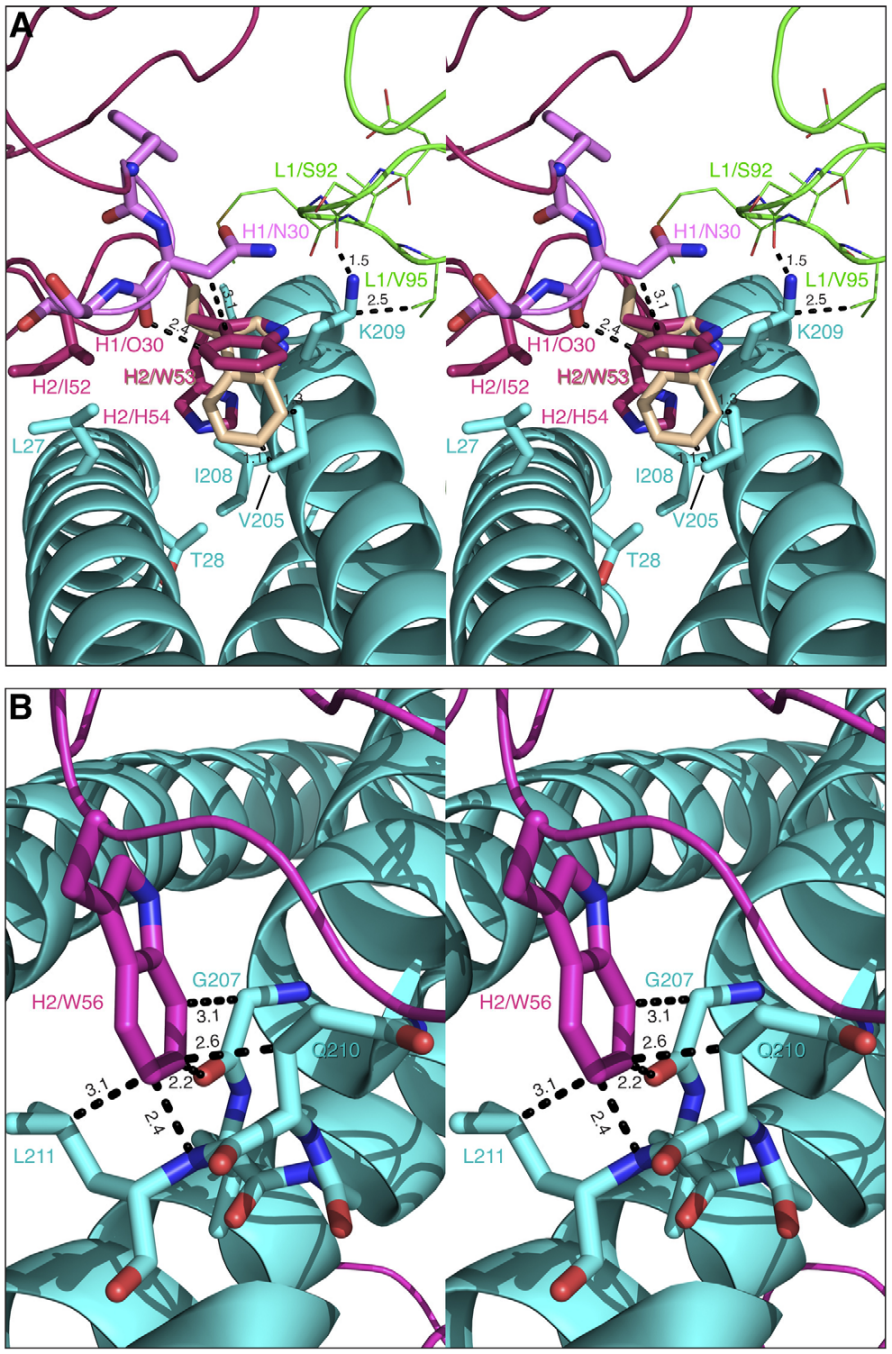
Modeled binding of the CDR-H2′s of Fabs 8065 and 8069 to 5-Helix. Helices of the N-HR trimer (cyan) are shown as a cartoon, with selected residues in stick representation. CDR-H2′s with selected residues shown as sticks are colored magenta. Short contacts are indicated by black dashed lines. (A) Model of the CDR-H2 of Fab 8065, with Trp-53 and His-54 shown in stick representation. The second rotamer of Trp-53 is shown in light brown, CDR-H1 and CDR-L1 with selected residues in stick are shown in pink and green, respectively. (B) A model of the CDR-H2 of Fab 8069, with Trp-56 shown in stick representation.

Fabs 8064 and 8065 are of interest since they display very different biological activity (neutralizing versus non-neutralizing, respectively), their affinities for 5-Helix differ by a factor of ∼30 (*K_D_* ∼5–10 nM versus ∼200 nM; unpublished data), and their CDR-H2 sequences differ at only two positions (Phe versus His at position 54, and Ser versus Gly at position 56). Both Fabs 8064 and 8065 have a bulky Trp at position 53 which likely necessitates some conformational changes, probably propagating to CDR-H1 and CDR-L1, to circumvent steric clash (Fig. 12A). However, the presence of a His at position 53 in Fab 8065 creates an unfavorable environment by introducing a polar residue into a hydrophobic pocket formed by *Leu-27* and *Thr-28* of helix *Na* and *Val-205* and *Ile-208* of helix *Nc*.

Fab 8069 also exhibits some unusual features since it differs from Fab 8062 by a single residue with a larger Trp substituting for a Phe at position 56. The presence of the bulky Phe-56 in Fab 8062 induced the changes in the conformation of CDR-H2 that were correlated in this study to the lack of biological activity of Fab 8062. Nevertheless, Fab 8069 has two-fold higher affinity for the N-HR mimetic N_CCG_-gp41 than Fab 8066 (∼7 versus ∼15 nM; [39]), a *K_D_* of 10-20 nM for binding to 5-Helix (unpublished data), and weak neutralization activity, roughly comparable to that of the parental Fab 3674 [39]. Modeling of the CDR-H2 of Fab 8069 based on that of the Fab 8062 complex shows that simple replacement of Phe-56 by a Trp results in numerous short contacts with residues of the *Nc* helix (Fig. 12B). Thus accommodation of a Trp at position 56 requires some structural rearrangements to occur within the gp41 bundle. However, since plasticity of the isolated N-HR trimer is likely to be greater than that of 5-Helix, such rearrangement could be readily achieved. While affecting the overall structure of a trimer, modeling suggests that, when bound, Trp-56 makes multiple tight contacts with an N-HR helix. This is reflected in the high affinity of Fab 8069 for the N-HR mimetic N_CCG_-gp41 [39]. However, shifts in the N-HR helices required to accommodate Trp-56 may prevent binding of more than one antibody molecule. This is in agreement with neutralization studies carried out in the presence of N_CCG_-gp41 which failed to demonstrate maximum antagonism at a molar ratio of Fab 8069 to N_CCG_-gp41 of 3∶1 (unpublished data), in contrast to the results with Fab 8066 [39] and even Fab 8064 (unpublished data) where maximum antagonism was observed at a 3∶1 molar ratio.

Based on the HIV-1/SIVcpz protein alignment from the 2009 Los Alamos Sequence Compendium [44], the N-HR epitope recognized by the two Fabs and the D5 antibody is highly conserved. The same or a closely overlapping epitope is also recognized by there other neutralizing antibodies directed against the N-HR trimer of gp41, namely DN9 and 8K8 [38]and HK20 [33]. Using the native gp160 numbering, the epitope comprises residues *560*, *563–565*, *567*, *568*, *570–577* and *579* of the N-HR (For the corresponding numbers of the residues in 5-helix in the structures of the complex, see Figure S1 in Supporting Information S1). Of these 15 residues, eight are absolutely conserved, and all substitutions involving the remaining seven residues are highly conservative (*E560N/Q*, *Q563R*, *H564Q/R*, *L565M*, *I573V*, *Q577R* and *R579Q*). Ten N-HR residues (*656*, *570–577* and *579*) interact with the CDR-H2, of which seven are absolutely conserved. In contrast, much more variability is seen for the seven C-HR residues within the epitope: only one residue is absolutely conserved (*E647*), three are subject to conservative substitutions (*D632E*, *H643Y* and *I646L*), and three (residues *629*, *636* and *640*) are not conserved at all. Only two C-HR residues, both of which are subject to conservative substitutions, interact with the heavy chain (CDR-H3). None of the C-HR residues that contact CDR-L1 are conserved, and two out of three that contact CDR-L2 are also not conserved. The two residues (*E647* and *H643Y*) contacting CDR-L2, however, are highly preserved. Thus, one can conclude that the broadly neutralizing properties of Fab 8066 largely originate from the conservation of the N-HR epitope across different strains and clades of HIV-1.

The remarkable difference in the neutralization activities of Fabs 8066 and 8062 [39], despite relatively small differences in the interactions with the antigen when compared with the D5 complex, would also suggest that adapting a productive conformation while binding to the pre-hairpin intermediate of gp41 is accompanied by structural rearrangements in the CDR-H2 region. It seems likely that the presence of two Phe residues at positions 54 and 56 of the CDR-H2 of Fab 8062 may result in a reduction in conformational plasticity of the CDR-H2 loop of Fab 8062 relative to that of Fab 8066, which results in the two Fabs adopting distinct CDR-H2 conformations upon binding 5-Helix. In both complexes every residue of the CDR-H2 loop is involved in interactions with 5-Helix, but topologically they are located very differently in relation to the body of the bundle (Figs. 2C, 5B and 5C). In particular, the CDR-H2 of Fab 8066 is buried in the body of the bundle, whereas in the case of Fab 8062 it is barely touching its surface (Figs. 5B, C). This difference also impacts the local conformation of 5-Helix, reflected by small but significant backbone atomic displacements in the region contacting the CDR-H2, as well as in immediately adjacent areas. The structural differences involving interactions of CDR-H2 with 5-Helix in the Fab 8066 and 8062 complexes are likely to perturb the lifetimes of the two complexes (as reflected in dissociation rate constants and hence affinity since the association rate constants are similar for the two complexes; unpublished data). Since the antibodies take the place of the *Cc* helix needed for completion of the structure of the 6-HB conformation of gp41 (Fig. 9), the lifetime of the complex will correlate with the ability of the antibody to prevent creation of a proper 6-HB needed for viral entry.

The different conformation and mode of interaction of the CDR-H2 of Fab 8066 and 8062 with 5-Helix also result in smaller propagated structural changes throughout the complex, which are likely to further contribute to the different binding affinities with 5-Helix. For example, there are a few additional interactions involving CDR-H3 (Fig. 6), CDR-L1 (Fig. 7A), CRD-L2 (Fig. 8), and CDR-L3 (Fig. 8) in the Fab 8066 complex compared to the Fab 8062 complex. Further, some of the interactions that are present in both complexes are stronger (i.e. shorter intermolecular contacts) in the Fab 8066 complex. Indeed, the gap volume index (which reflects the tightness of the interfacial packing [43]) is significantly smaller in the Fab 8066 complex (2.3 Å) than in the Fab 8062 complex (2.6 Å).

Although Fabs 8066 and 8062 are both able to bind to the exposed N-HR trimer with similarly high affinity [39], their ability to bind to the 5-Helix construct differs by about 2 orders of magnitude, and that difference is reflected in their neutralization activity (Table 1). This observation leads us to two conclusions. First, the interactions of the *Ca* helix of 5-Helix with the CDR-H3 (Fig. 6), CDR-L2 (Fig. 8) and CDR-L3 (Fig. 8) loops may modulate the affinity of Fabs 8066 and 8062. Second, the assay (involving a solution equilibrium titration in conjunction with an electrochemiluminescence-based affinity measurement) used to determine *K_D_* values for the binding of Fabs to the N-HR trimer mimetic N_CCG_-gp41 reported by Gustchina et al. [39] reflect the binding of only a single Fab to N_CCG_-gp41, and hence do not report on any possible negative cooperativity arising from steric clash between neighboring Fabs bound to the N-HR trimer (cf. Fig. 11).

A key feature of fusion inhibitors that target the N-HR trimer, including presumably antibodies directed against the N-HR trimer, is that deactivation of gp41 *in vivo* is a slow reversible process that is dependent on chemokine receptor binding to Env, and that the exposed N-HR trimer remains accessible to inhibitors until the final conformational changes in gp41 that lead to the formation of the 6-HB have taken place [31]. This suggests that inhibition of fusion will be most effective when two or more Fabs are bound to the exposed N-HR trimer of the pre-hairpin intermediate of gp41. Since multiple Fabs/antibodies bound to the N-HR trimer are unlikely to dissociate simultaneously, the probability that at least one antibody is bound to the N-HR trimer at all times will be increased. Our modeling suggests that three molecules of the most potent neutralizing Fab in our series, Fab 8066, can readily bind to the N-HR trimer without any steric clashes between adjacent Fab molecules (Figs. 10 and 11). In contrast, as a consequence of the different mode of binding of the CDR-H2 to the hydrophobic pocket on the surface of the N-HR trimer, binding of three molecules of Fab 8062 to the N-HR trimer may result in steric clash between adjacent Fab molecules involving the CDR-H2, CDR-L1, CDR-L3 loops and loop 71–78 (Fig. 11). It seems likely in the light of the current structural data, modeling results and neutralization properties of our Fab series [39], that neutralization is dependent not only on tight binding of a single Fab to the N-HR trimer but also on the ability to bind multiple Fabs to a single N-HR trimer at a preliminary step of the fusion process.

## Materials and Methods

### Expression and purification of 5-Helix and Fabs 8066 and 8062

The plasmid construct to express 5-Helix fused to a C-terminal His-tag was generously provided by Michael Root (Thomas Jefferson University). A stop codon was engineered preceding the His-tag using the Quik-Change mutagenesis protocol (Stratagene, La Jolla, CA) to express a tag-less version of 5-Helix. 5-Helix was expressed in *E. coli*, purified from the insoluble fraction under denaturing conditions on a Superdex-200 column, and then subjected to reverse-phase HPLC. The protein was dialyzed against 50 mM sodium formate, pH 3, concentrated to ∼10 mg/ml and stored at 4°C.

Fabs 8066 and 8062 were initially expressed and purified as described previously. Further purification was carried out by size-exclusion chromatography on a Superose-12 column (16×60 cm, GE HealthCare) equilibrated in 10 mM Tris-HCl, pH 7.5, 150 mM NaCl. Peak fractions were pooled and concentrated to ∼2 mg/ml and stored frozen. The D5 monoclonal antibody in IgG and Fab formats was generously provided by Joseph Joyce (Merck Research Laboratories).

Liquid chromatography-mass spectrometry/mass spectrometry analysis [45] of endoprotease (trypsin, Lys-C, Glu-C, chymotrypsin) digested Fabs either as liquid samples [46] or after separation by SDS/PAGE [47] was used to provide partial sequence information of regions of the engineered Fabs prior to obtaining the full sequences from AbD Serotec.

### Assessment of the Fab/5-Helix complexes by SMR

**S**ize exclusion chromatography with detection by **M**ultiangle light scattering (DAWN EOS, Wyatt Technology Inc., Santa Barbara, CA) and **R**efractive index (OPTILAB DSP, Wyatt Technology) (SMR) was used for mass analysis of 5-Helix in complex with Fab 8066 and to optimize conditions for the large-scale preparation of complexes for crystallization. 5-Helix (103 µg) and Fab 8066 (106 µg) before and after mixing with each other (∼50 µg each giving a molar ratio of 1∶2 Fab 8066 to 5-Helix) in a final volume of 150 µl were loaded on Superdex-75 column (1×30 cm) equilibrated in 1x PBS at a flow rate of 0.5 ml at room temperature. Molecular masses were calculated using the Astra software provided with the instrument.

### Preparation of large-scale antigen-antibody complexes

Preparation of the Fab 8066/5-Helix complexes for crystallization was carried out by slowly mixing 64 µl of 5-Helix (575 µg corresponding to a 1.5-fold molar excess over Fab) with 1.5 ml of Fab (∼ 0.8 mg) solution kept in 10 mM Tris-HCl, pH 7.5 and 150 mM NaCl and separating the complex on a Superose-12 column (16×60 cm) in the same buffer. Because of the lower affinity of Fab 8062 for 5-Helix, a 3-4 fold higher protein concentration of the Fab 8062/5-Helix complex was maintained prior to subjecting the complex for fractionation on the column. The complexes were concentrated to a final concentration of ∼10 mg/ml and stored at 4°C.

### Isothermal Titration Calorimetry (ITC)

ITC measurements were performed using an ITC200 titration calorimeter (Microcal Inc.) at 28°C. The stock solution (∼10 mg/ml) of 5-Helix in 50 mM sodium formate, pH 3, was diluted to about 0.5 mg/ml in 25 mM Tris-HCl, pH 7.5, 150 mM NaCl, dialyzed against 10 mM Tris, pH 7.5, 150 mM NaCl and concentrated to ∼5 mg/ml. Antibody solutions (5–15 µM) in 10 mM Tris, pH 7.5, 150 mM NaCl kept in the calorimetric cell were titrated against 10 and 20 fold higher concentration of 5-Helix for monovalent Fabs 8062, 8066 and D5, respectively. The data were analyzed using the Origin software provided with the instrument.

### HIV-1 neutralization assay

The cell lines and molecular clones employed were identical to those described in our previous publication [39]. Preparation of Env-pseudotyped HIV-1 and Env-pseudotyped HIV neutralization assays (employing TZM-b1 indicator cells that constitutively express CD4, CCR4 and CXCR4) were carried out exactly as described previously [39].

### Crystallographic procedures

Crystallization of the Fab 8066/5-Helix and Fab 8062/5-Helix complexes was carried out by the hanging drop, vapor diffusion method. The Fab 8066/5-Helix complex was concentrated to 7 mg/ml in 10 mM Tris buffer at pH 8.0, also containing 0.15 M NaCl. The reservoir solution contained 1.2 M dibasic ammonium phosphate in 0.1 M HEPES buffer at pH 8.0. Each drop contained 2 µl of protein samples and 2 µl of reservoir solution. The crystals grew to the maximum size of ∼0.2 mm in 4 to 5 days. The Fab 8062/5-Helix complex was treated in a similar way, with the exception that the well solution contained 14% polyethylene glycol 10K, 0.1 M ammonium sulfate, and 5% ethylene glycol. Diffraction data for both complexes were collected using synchrotron radiation at the SER-CAT ID-22 beamline at the APS, Argonne National Laboratory, from one crystal each. The resolution of the data for the Fab 8066/5-Helix complex and Fab 8062/5-Helix complex was 2.05 and 2.5 Å, respectively. The structure of the Fab 8066/5-Helix complex was solved by molecular replacement with the program PHASER [48], using the coordinates of the D5/5-Helix complex ([41] PDB ID 2CMR) as the search model. The structure was completed through a number of cycles of refinement with REFMAC5 [49] and rebuilding with COOT [50]. The refined structure of the Fab 8066/5-Helix complex was used as the search model to solve the structure of the Fab 8062/5-Helix complex, with similar procedures used for subsequent refinement and model building. The statistics for data collection and structure refinement are listed in Table 2. The coordinates and structure factors for the Fab 8066/5-Helix and Fab 8062/5-Helix complexes have been deposited in the Protein Data Bank with IDs 3MA9 and 3MAC, respectively.

## Supporting Information

Supporting Information S1 Figures and Tables providing a reference for the residue numbering in 5-helix (Figure S1), a detailed list of antigen-antibody contacts (Table S1), and a comparison of CDR-H2 sequences of neutralizing and non-neutralizing antibodies (Table S2).
(0.11 MB PDF)
