# Supplementary material for: Structural Basis of HIV-1 Neutralization by Affinity Matured Fabs Directed against the Internal Trimeric Coiled-Coil of gp41
Source: PLoS Pathog. 2010 Nov 11;6(11):e1001182. doi: 10.1371/journal.ppat.1001182 (PMC2978731; doi:10.1371/journal.ppat.1001182)
Supplement: Supporting Information S1 — Figures and Tables providing a reference for the residue numbering in 5-helix (Figure S1), a detailed list of antigen-antibody contacts (Table S1), and a comparison of CDR-H2 sequences of neutralizing and non-neutralizing antibodies (Table S2). (0.11 MB PDF) [file ppat.1001182.s001.pdf]

SUPPORTING INFORMATION

Structural Basis of HIV-1 Neutralization by Affinity Matured Fabs Directed Against the Internal Trimeric Coiled-Coil of gp41

Elena Gustchina[1], Mi Li[2,3], John M. Louis[1], D. Eric Anderson[4], John Lloyd[4], Christian Frisch[5], Carole A. Bewley[6], Alla Gustchina[2]*, Alexander Wlodawer[2]*, and G. Marius Clore[1]*

[1]Laboratory of Chemical Physics, Building 5, National Institute of Diabetes and Digestive and Kidney Diseases, National Institutes of Health, Bethesda, MD 20892-0520

[2]Protein Structure Section, Macromolecular Crystallography Laboratory, National Cancer Institute, Frederick, Maryland 21702-1201

[3]Basic Research Program, SAIC-Frederick, Frederick, MD 21702-1201

[4]Proteomics and Mass spectrometry Facility, Building 8, National Institute of Diabetes and Digestive and Kidney Diseases, National Institutes of Health, Bethesda, MD 20892-0820

[5]AbD Serotec, MorphoSys AG, Lena-Christ-Strasse 48, 82152 Martinsried, Germany

[6]Laboratory of Biorganic Chemistry, Building 8, National Institute of Diabetes and Digestive and Kidney Diseases, National Institutes of Health, Bethesda, MD 20892-0820

Figure S1 Comparison of residue numbering in 5-Helix as introduced here (the range is for the complex with Fab 8066) with the numbering in the structure of the D5 complex, as well as in the sequence of native HIV-1 gp160. (The helices are highlighted by the gray boxes; and residues of 5-helix not visible in the electron density map of the Fab8066/5-helix complex are shown in small letters).

Sequence		M	Q	L	L	S	G	I	V	Q	Q	Q	N	N	L	L	R	A	I	E	A	Q	Q	H	L	L	Q	L	T	V	W	G	I	K	Q	L	Q	A	R	I	L	A	G	g	s	g	g
3MA9	*Na*	1	2	3	4	5	6	7	8	9	10	11	12	13	14	15	16	17	18	19	20	21	22	23	24	25	26	27	28	29	30	31	32	33	34	35	36	37	38	39	40	41	42	43	44	45	46
2CMR		176[G]177[G]	178	179	180	181	182	183	184	185	186	187	188	189	190	191	192	193	194	195	196	197	198	199	200	201	202	203	204	205	206	207	208	209	210	211	212	213	214	215	216						
gp160			543	544	545	546	547	548	549	550	551	552	553	554	555	556	557	558	559	560	561	562	563	564	565	566	567	568	569	570	571	572	573	574	575	576	577	578	579	580	581	582					

Sequence		H	T	T	W	M	E	W	D	R	E	I	N	N	Y	T	S	L	I	H	S	L	I	E	E	S	Q	N	Q	Q	E	K	N	E	Q	E	L	L	E	g	s	s	g				
3MA9	*Ca*	47	48	49	50	51	52	53	54	55	56	57	58	59	60	61	62	63	64	65	66	67	68	69	70	71	72	73	74	75	76	77	78	79	80	81	82	83	84	85	86	87	88				
2CMR					138	139	140	141	142	143	144	145	146	147	148	149	150	151	152	153	154	155	156	157	158	159	160	161	162	163	164	165	166	167	168	169	170	171	172								
gp160		625	626	627	628	629	630	631	632	633	634	635	636	637	638	639	640	641	642	643	644	645	646	647	648	649	650	651	652	653	654	655	656	657	658	659	660	661	662								

Sequence		G	Q	L	L	S	G	I	V	Q	Q	Q	N	N	L	L	R	A	I	E	A	Q	Q	H	L	L	Q	L	T	V	W	G	I	K	Q	L	Q	A	R	I	L	a	g	g	s	g	g
3MA9	*Nb*	89	90	91	92	93	94	95	96	97	98	99	100	101	102	103	104	105	106	107	108	109	110	111	112	113	114	115	116	117	118	119	120	121	122	123	124	125	126	127	128	129	130	131	132	133	134
2CMR		89	90	91	92	93	94	95	96	97	98	99	100	101	102	103	104	105	106	107	108	109	110	111	112	113	114	115	116	117	118	119	120	121	122	123	124	125	126	127	128						
gp160			543	544	545	546	547	548	549	550	551	552	553	554	555	556	557	558	559	560	561	562	563	564	565	566	567	568	569	570	571	572	573	574	575	576	577	578	579	580	581	582					

Sequence		h	T	T	W	M	E	W	D	R	E	I	N	N	Y	T	S	L	I	H	S	L	I	E	E	S	Q	N	Q	Q	E	K	N	E	Q	E	L	L	E	g	s	s	g				
3MA9	*Cb*		136	137	138	139	140	141	142	143	144	145	146	147	148	149	150	151	152	153	154	155	156	157	158	159	160	161	162	163	164	165	166	167	168	169	170	171	172	173	174	175	176				
2CMR				49	50	51	52	53	54	55	56	57	58	59	60	61	62	63	64	65	66	67	68	69	70	71	72	73	74	75	76	77	78	79	80	81	82	83									
gp160		625	626	627	628	629	630	631	632	633	634	635	636	637	638	639	640	641	642	643	644	645	646	647	648	649	650	651	652	653	654	655	656	657	658	659	660	661	662								

Sequence		G	Q	L	L	S	G	I	V	Q	Q	Q	N	N	L	L	R	A	I	E	A	Q	Q	H	L	L	Q	L	T	V	W	G	I	K	Q	L	Q	A	R	I	L	a	g	g	s	g	g
3MA9	*Nc*	177	178	179	180	181	182	183	184	185	186	187	188	189	190	191	192	193	194	195	196	197	198	199	200	201	202	203	204	205	206	207	208	209	210	211	212	213	214	215	216	217	218	219	220	221	222
2CMR		1[M]	2	3	4	5	6	7	8	9	10	11	12	13	14	15	16	17	18	19	20	21	22	23	24	25	26	27	28	29	30	31	32	33	34	35	36	37	38	39	40	41					
gp160			543	544	545	546	547	548	549	550	551	552	553	554	555	556	557	558	559	560	561	562	563	564	565	566	567	568	569	570	571	572	573	574	575	576	577	578	579	580	581	582					

Residue numbering of 5-Helix

The numbering of 5-Helix given here is the correct sequential numbering of the polypeptide chain of 5-Helix from its N to C-terminus. The ordering of the N-HR and C-HR helices is N_a-C_a-L-N_b-C_b-L-N_c, where L is a five-residue linker. In the context of the trimeric 6-HB structure of gp41, N_a and C_a, N_b and C_b, and N_c and C_c belong to three separate subunits: thus the N_a/C_a, N_b/C_b and N_c/C_c interactions are intrasubunit in the 6-HB gp41 trimer [S1]. To obtain the correct numbering of 5-Helix in the deposited 2CMR coordinates of the D5/5-helix complex [S2], residues 1-40 in 2CMR need to be renumbered 177-216 (i.e. add 176, and the N-terminal Met in the 2CMR coordinates is actually Gly177); residues 176-218 become residues 0-42 (i.e. subtract 176, and the two glycines at positions 176 and 177 become an N-terminal Met at position 1); residues 135 to 172 become residues 47-84 (i.e. subtract 88); residues 89-128 are unchanged; and residues 48-84 become residues 136-172 (i.e. add 88). In essence, the ordering of the helices in 2CMR was permuted such that N_a(2CMR)→Nc, C_a(2CMR)→C_b, N_b(2CMR)→N_b, C_b(2CMR)→C_a, and N_c(2CMR)→N_a.

S1. Caffrey M, Cai M, Kaufman J, Stahl SJ, Wingfield PT, et al. (1998) Three-dimensional solution structure of the 44 kDa ectodomain of SIV gp41. EMBO J 17: 4572-4584.

S2. Luftig MA, Mattu M, Di Giovine P, Geleziunas R, Hrin R, et al. (2006) Structural basis for HIV-1 neutralization by a gp41 fusion intermediate-directed antibody. Nature Struct Mol Biol 13: 740-747.

Table S1 A detailed list of antigen-antibody contacts in the Fab 8066/5-Helix, Fab 8062/5-Helix, and D5/5-Helix complexes.

CDR	8066	8062	D5
L1<br>Hydrophobic Contacts	P27 to *N58* (*Ca*)<br>Y31 to *W30* (*Na*)	Y31 to *W30* (*Na*)	Y30 to *N58* (*Ca*)<br>W32 to *H65* (*Ca*)<br>W32 to *T61* (*Ca*)
Polar Interactions	E30 to *S62* (*Ca*)<br>Y31 to *K33* (*Na*)<br>Y31 to *N58* (*Ca*)	E30 to *H65* (*Ca*, ionic pair 2.5 A)<br>Y31 to *K33* (*Na*, weak)<br>Y31 to *N58* (*Ca*)	W32 to *N58* (*Ca*, strong HB[a] 2.8 A)
L2	N52 to *H65* (*Ca*, polar bridge through H_2O)<br>N52 to *E69* (*Ca*, polar bridge through H_2O)<br>Y48 to N103 (CDR H3) to *H65* (*Ca*, polar bridge through H_2O)		K50 to *H65* (*Ca*)
L3<br>Hydrophobic Contacts	M93 to *M51* (*Ca*)<br>M93 to *D54* (*Ca*, 2 contacts)<br>M93 to *K33* (*Na*)<br>V95 to *W30* (*Na*, 3 contacts)<br>V95 to *Q34* (*Na*)<br>V95 to *K33* (*Na*, 3 contacts)	M93 to *N54* (*Ca*, 2 contacts)<br>V95 to *W30* (*Na*, 4 contacts+1 weak)<br>V95 to *Q34* (*Na*)<br>V95 to *K33* (*Na*, 2 contacts)	Y94 to *Q34* (*Na*, 2 contacts)<br>Y94 to *K33* (*Na*)<br>Y94 to *W30* (*Na*, weak)
Polar Interactions	S92 to *N58* HB (*Ca*)<br>S92 to *K33* HB (*Na*)<br>S92 to *D54* two HB (*Ca*)	S92 to *K33* HB (*Na*, weak)<br>S92 to *D54* two HB (*Ca*)	S92 to *K33* HB (*Na*)<br>S92 to *N54* (*Ca*, polar bridge through H_2O)

H1<br>Hydrophobic Contacts<br><br><br>Polar Interactions	S31 to *L27* (*Na*)<br>A33 to *L27* (*Na*, weak)<br><br><br>S31 to *H23* HB (*Na*)	S31 to *L27* (*Na*)<br>A33 to *L27* (*Na*, weak)<br><br><br>S31 to *H23* HB (*Na*)	S31 to *L27* (*Na*)<br>A33 to *L27* (*Na*)<br>A33 to *W30* (*Na*)<br><br>S31 to *H23* (*Na*, strong HB 2.5 Å)
H2<br>Hydrophobic Contacts<br><br><br>Polar Interactions	I52 to *W30* (*Na*, 2 contacts)<br>I53 to *V205* (*Nc*)<br>I53 to *L24* (*Na*)<br>F54 to *V205* (*Nc*)<br>F54 to *I208* (*Nc*)<br>F54 to *K209* (*Nc*)<br>F54 to *G31* (*Na*)<br>G55 to *Q212* (*Nc*)<br>T56 to *L35* (*Na*)<br><br>T56 to *Q34* HB (*Na*)<br>T57 to *R38* (*Na*, strong HB 2.7 Å)<br>G55 to *Q212* HB (*Nc*)<br>N58 to *Q34* HB (*Na*)	I52 to *W30* (*Na*, 1 weak)<br>L53 to *L24* (*Na*)<br>F54 to *V205* (*Nc*)<br>F54 to *I208* (*Nc*)<br>F54 to *K209* (*Nc*)<br>F54 to *G31* (*Na*)<br>F56 to *Q212* (*Nc*)<br>F56 to *L35* (*Na*)<br>F56 to *Q34* (*Na*)<br>V58 to *Q34* (*Na*)<br><br>A57 to *R38* (*Na*, strong HB 2.7 Å)	I52 to *W30* (*Na*)<br>F54 to *V205* (*Nc*)<br>F54 to *I208* (*Nc*)<br>F54 to *K209* (*Nc*)<br>F54 to *G31* (*Na*)<br>G55 to *Q212* (*Nc*)<br>T56 to *L35* (*Na*)<br><br>T56 to *Q34* HB (*Na*)
H3<br>Hydrophobic Contacts<br><br><br>Polar Interactions	Y98 to *W30* (*Na*, 2 contacts)<br>F99 to *H23* (*Na*)<br>F99 to *W30* (*Na*)<br>F99 to *L27* (*Na*)<br>Y102 to *I68* (*Ca*)<br>Y102 to *E19* (*Na*)<br>Y102 to *Q22* (*Na*, 2 contacts)<br>Y102 to *Q26* (*Na*)<br>Y105 to *W30* (*Na*)<br><br>Y98 to *W30* HB (*Na*)<br>Y102 to *H65* (*Ca*, strong HB 2.8 Å)<br>Y102 to *Q26* (*Na*, strong HB 2.8 A)	Y98 to *W30* (*Na*, 2 contacts, weaker)<br>F99 to *H23* (*Na*)<br>F99 to *W30* (*Na*)<br>F99 to *L27* (*Na*)<br>Y102 to *I68* (*Ca*)<br>Y102 to *E19* (*Na*)<br>Y102 to *Q22* (*Na*, 2 contacts)<br>Y102 to *Q26* (*Na*)<br>Y105 to *W30* (*Na*)<br><br>Y98 to *W30* (*Na*, weak HB 3.5 Å)<br>Y102 to *Q26* (*Na*, weak HB 3.4 A)	P97 to *H23* (*Na*)<br>P97 to *L27* (*Na*)<br>P97 to *W30* (*Na*)<br>L100 to *W30* (*Na*)<br><br>T98 to *H23* (*Na*, strong HB 2.7 Å)

[a]HB, hydrogen bond.

Table S2. CDR-H2 sequences of antibodies directed against the N-HR trimer of gp41

Antibody	CDR-H2																
3674	G	I	I	P	I	F	G	M	A	N	Y	A	Q	K	F	Q	G
8059	S	I	I	P	L	F	G	T	T	N	Y	A	Q	K	F	Q	G
8060	S	I	I	P	I	F	G	S	T	N	Y	A	Q	K	F	Q	G
8061	S	I	I	P	M	M	G	S	T	N	Y	A	Q	K	F	Q	G
8062	S	I	I	P	L	F	G	F	A	V	Y	A	Q	K	F	Q	G
8063	S	I	I	P	V	I	G	S	T	N	Y	A	Q	K	F	Q	G
8064	S	I	I	P	W	F	G	S	T	N	Y	A	Q	K	F	Q	G
8065	S	I	I	P	W	H	G	G	T	N	Y	A	Q	K	F	Q	G
8066	S	I	I	P	I	F	G	T	T	N	Y	A	Q	K	F	Q	G
8068	S	I	I	P	L	M	G	T	T	N	Y	A	Q	K	F	Q	G
8069	S	I	I	P	L	F	G	W	A	N	Y	A	Q	K	F	Q	G
D5	G	I	I	P	I	F	G	T	A	N	Y	A	Q	A	F	Q	G
DN9	G	L	I	P	L	F	E	T	T	N	Y	A	Q	N	F	Q	G
8k8	Y	I	D	P	I	F	G	R	A	H	Y	A	R	W	V	N	D
residue	50	51	52	52a	53	54	55	56	57	58	59	60	61	62	63	64	65

Residues 53, 54 and 56 are highlighted since these sites represent the most frequently mutated residues and appear to be the primary determinants of neutralization activity.
